# Facial Temperature Responses to Ostracism in Women: Exploring Nasal Thermal Signatures of Different Coping Behaviors

**DOI:** 10.1111/psyp.70081

**Published:** 2025-06-08

**Authors:** Anneloes Kip, Thorsten M. Erle, Ilja van Beest

**Affiliations:** ^1^ Department of Social Psychology, Tilburg School of Social and Behavioral Sciences Tilburg University Tilburg the Netherlands; ^2^ Department of Methodology and Statistics, Faculty of Health, Medicine and Life Sciences Maastricht University Maastricht the Netherlands

**Keywords:** coping behavior, ostracism, thermal infrared imaging

## Abstract

Ostracism (feeling ignored and excluded) triggers psychophysiological responses associated with distress. We investigated different coping responses after ostracism and explored whether these were preceded by unique facial thermal signatures, reflecting autonomic nervous system (ANS) activity. Using thermal infrared imaging, we recorded facial cutaneous temperature variations in female participants (*N* = 95) experiencing inclusion and ostracism using hypothetical Cyberball games. Coping after ostracism was assessed during a hypothetical Allocation Game, where participants could do nothing (withdrawal), reduce (antisocial), or increase (prosocial) the hypothetical earnings of their ostracizer. Contrary to expectations, most participants chose to withdraw (52%), with fewer opting for antisocial responses (30%) or prosocial responses (18%) after ostracism. Results from linear mixed‐effects modeling revealed that substantial temperature variability occurred only in the nose region of the face. Both ostracism and inclusion showed a decrease in nasal temperature relative to baseline, but the average drop was greater during inclusion, suggesting stronger ANS activation during inclusion rather than ostracism. Crucially, exploratory findings showed that only participants who responded antisocially after ostracism exhibited steeper decreases in nasal temperature during ostracism compared to inclusion. This pattern suggests greater physiological reactivity among antisocial responders, particularly in contrast to those who chose to withdraw. Future research should integrate thermal imaging with other physiological measures and strengthen ostracism manipulations to understand the relationship between thermal responses and different coping behaviors.

## Impact Statement

Our study challenges assumptions about ostracism's psychophysiological effects, finding no heightened autonomic activation in facial temperature during exclusion. However, distinct nasal thermal patterns emerged based on coping behaviors. These results urge caution in interpreting thermal imaging as a marker of distress and highlight its potential for differentiating post‐ostracism responses.

## 1 Introduction

Ostracism, defined as being ignored and excluded by others (Williams 2009), is a distressing experience that negatively affects people's emotions and psychological needs (Williams 2009; Ren et al. 2018). To cope, targets may exhibit different coping strategies, typically classified as antisocial, prosocial, and withdrawal responses (Ren et al. 2018; Smart Richman and Leary 2009; Williams 2009). Furthermore, ostracism is known to trigger neurological and physiological reactions associated with threat detection, psychological distress, and pain (e.g., Ferris 2019; Eisenberger et al. 2007; Eisenberger and Lieberman 2004; Gunther Moor et al. 2010; Kelly et al. 2012; Sijtsema et al. 2011; Sleegers et al. 2017). These reactions are closely tied to the autonomic nervous system (ANS), particularly through the activation of the sympathetic nervous system (SNS), which readies the body for a fight‐or‐flight response and influences cardiovascular, metabolic, and respiratory functions (e.g., Cannon 1915; Goldstein 2021). Despite the abundance of research on psychophysiological responses to ostracism, our understanding of how these physiological changes align with various coping behaviors remains limited. In the present study, we investigated the occurrence of different coping responses after ostracism and explored whether these were preceded by unique facial thermal signatures, used as a proxy for ANS activity.

### 1.1 Coping Behaviors in Response to Ostracism: Prosocial, Antisocial, and Withdrawal Behaviors

According to the Temporal Need Threat Model (TNTM; Williams 2009; Williams and Nida 2011; Ren et al. 2018) and the Multimotive Model of Interpersonal Rejection (Smart Richman and Leary 2009), targets may cope with the detrimental consequences of ostracism through prosocial, antisocial, and withdrawal strategies. Prosocial responses, which include cooperation and behaviors aimed at benefiting or helping others, are thought to promote (re‐)inclusion or affiliation, thereby restoring a sense of belonging and self‐esteem (Smart Richman and Leary 2009; Williams 2009). Conversely, antisocial responses may manifest as aggressive actions or behaviors that harm others. Such responses are thought to be motivated by a desire for retaliation or punishment against the source of ostracism, as a means to regain control (Ren et al. 2018; Williams 2009). Withdrawal responses to ostracism are characterized by disengagement from interaction and avoidance of the source of ostracism and may be driven by a need for solitude or to (mentally) escape from the painful experience (Smart Richman and Leary 2009; Ren et al. 2016, 2021; Wesselmann et al. 2015).

Different coping strategies are likely to emerge following ostracism, each with its own set of costs and benefits. Following ostracism, withdrawal responses are particularly appealing because they minimize costs and avoid conflict, providing immediate emotional relief (Lazarus and Folkman 1984; Roth and Cohen 1986; Sunami et al. 2019; Williams 2007). Furthermore, while prosocial responses are generally favorable because they strengthen social bonds and meet the fundamental need to belong, they are also considered riskier, as attempts to reconcile or cooperate may lead to further rejection or exploitation (Axelrod 1980; Maner et al. 2007). Prior theorizing (DeWall and Richman 2011) suggests that when given an opportunity to reconnect, targets of ostracism are more likely to cope in a prosocial manner rather than antisocially, especially when such options carry no immediate personal costs. Conversely, antisocial responses may be the least appealing due to their high risks and potential for conflict (Baumeister and Vohs 2004; Baumeister and Leary 1995; Ren et al. 2018; Williams 2007).

To illustrate, Kip et al. (2025, Study 3) found that ostracized individuals prioritized prosocial responses when all coping options were non‐costly and were least likely to choose antisocial responses. Participants could choose to either increase outcomes (prosocial), decrease outcomes (antisocial), or do nothing (withdrawal) toward their ostracizer. However, it remains unclear whether these findings generalize across different contexts, particularly given that few studies have explicitly examined the selection of coping responses when these three options (prosocial, antisocial, and withdrawal) are available simultaneously. Furthermore, little is known about how physiological mechanisms, such as ANS activity, relate to these coping decisions when multiple options are available. The current study addresses this gap by examining whether participants again favored prosocial behavior over withdrawal and antisocial behavior. Additionally, we explored how different coping responses after ostracism were associated with ANS activity.

### 1.2 Exploring Autonomic Responses to Ostracism Through Thermal Infrared Imaging

One way in which researchers measure ANS activity in response to emotional situations and psychological distress is by thermal infrared imaging (Kosonogov et al. 2017). Thermal infrared imaging captures subtle changes in skin temperature resulting from alterations in peripheral blood flow, due to changes in SNS activity. Compared to other physiological measurements, this method offers the additional benefits of being relatively non‐invasive and contact‐free (Ioannou et al. 2014; Van Beest and Sleegers 2019). In the field of psychology, thermal infrared imaging is used for a variety of research topics, including, but not limited to, the classification and assessment of emotions (e.g., Cruz‐Albarran et al. 2017; Jamal and Kamioka 2019; Khan et al. 2006, 2009; Nhan and Chau 2009; Kosonogov et al. 2017) as well as the examination of fear and stress‐related responses (e.g., Sonkusare et al. 2019) and social interactions (for a brief overview see: Cardone and Merla 2017). These studies have examined cutaneous temperature changes in different regions of the body, including the fingertips but primarily the face.

Research on facial cutaneous temperature changes in response to stress and emotional experiences has examined various regions of interest (ROIs), including the nose, cheeks, forehead, periorbital region, perioral region, and chin (e.g., Cho et al. 2019; Cruz‐Albarran et al. 2017; Rimm‐Kaufman and Kagan 1996; Sonkusare et al. 2019). Studies suggest that increased ANS activity can lead to either an increase or decrease in facial temperature, depending on local anatomical factors such as muscle distribution and the presence of adrenergic receptors in different ROIs (Bentsianov and Blitzer 2004; Derakhshan et al. 2019). For example, the cheeks may show an increase in temperature due to vasodilation, as seen in blushing due to embarrassment (Drummond et al. 2003; Ioannou et al. 2017), while the nose typically shows a decrease in temperature due to vasoconstriction, a response commonly associated with stress or negative emotional states (Cho et al. 2019; Genno et al. 1997; Kuraoka and Nakamura 2011; Nakayama et al. 2005; Salazar‐López et al. 2015). However, research specifically examining facial temperature responses to ostracism remains limited. To address this gap and ensure comprehensive analysis, we included a wide range of facial ROIs in our study.

Previous studies investigating the influence of ostracism on thermal responses yield mixed findings regarding the direction of temperature changes, depending on the specific ROI investigated. Being ostracized (compared to being included) in a virtual ball‐tossing game (i.e., the Cyberball paradigm; Williams and Jarvis 2006) led to a reduction in temperature in the extremities of the body (i.e., fingertip; IJzerman et al. 2012) over time. In contrast, ostracism was associated with elevated temperatures in the face, for example, at the tip of the nose (Paolini et al. 2016; Mazzone et al. 2017), and the perioral regions (only for women: Paolini et al. 2016) or the periorbital area (Ponsi et al. 2019). Another aim of this study was to replicate the effects of ostracism on facial cutaneous temperature, using a similar induction of ostracism, assessing a broad set of facial ROIs, and a larger sample. Given the conflicting results of previous studies on ostracism and other negative emotional events on facial temperature changes (i.e., heating up or cooling down), and the observed variations across facial ROIs, we refrained from making specific directional predictions. Instead, we hypothesized that ostracism (compared to inclusion) would lead to bigger changes in facial cutaneous temperature, given its association with stronger ANS activation. We additionally hypothesized that this difference in temperature changes would become more pronounced over time, as experiences of inclusion and ostracism gradually intensify during the Cyberball paradigm (also see IJzerman et al. 2012). We reasoned that participants need time to fully perceive and process their inclusion or exclusion status, with these experiences progressively unfolding and reinforcing themselves until the game concludes.

Besides the influence of ostracism on facial cutaneous temperature changes, we also aimed to examine whether these physiological responses are linked to specific coping behaviors after ostracism. Previous research on physiological responses to threats or emotional events has shown that the body triggers reactions to prepare itself, mobilizing energy for a “fight or flight” response (Eisenberger 2013). This increased SNS activity is typically associated with coping behaviors that involve both approaching and active avoidance of threats (Beauchaine 2001; Gray and McNaughton 2000). Most studies on neurophysiological responses to ostracism have not examined the direct link between physiological changes and different coping strategies or have done so focusing on a single behavioral response option (e.g., neural correlates rejection‐aggression link: Chester et al. 2014, 2018; social‐reconnection hypothesis: Masten et al. 2011). For example, Ponsi et al. (2019) found that periorbital temperature changes during ostracism differed between psoriasis patients and healthy controls in how they related to trust (i.e., monetary investment towards an unfamiliar player), although no significant relationship was found within either group. However, the study did not explore conditions that might examine facial thermal responses in relation to alternative coping strategies, such as aggression or withdrawal. Thus, we extend these findings by exploring whether different coping strategies following ostracism are preceded by unique thermal patterns in the face.

### 1.3 The Current Research

We examined different coping responses after ostracism and explored whether these were preceded by unique facial thermal signatures. To align with previous research (IJzerman et al. 2012; Mazzone et al. 2017; Paolini et al. 2016; Ponsi et al. 2019), we used a hypothetical Cyberball game (Williams and Jarvis 2006) and thermal infrared imaging to assess ANS activation during imagined experiences of inclusion and ostracism. We hypothesized that prosocial responses after ostracism would be more frequent compared to antisocial responses (H1) and compared to withdrawal responses (H2).^1^ We additionally hypothesized that participants would show greater ANS activation during ostracism compared to inclusion experiences (H3), marked by bigger changes in facial cutaneous temperature, and that this difference would increase over time (H4). We additionally explored the relationship between facial temperature changes and subsequent coping behaviors without a specific a priori hypothesis. The preregistration, anonymized data, code, power simulation, materials, and supporting information for this research are available at the Open Science Framework (OSF): https://osf.io/mhc67/.

## 2 Method

### 2.1 Sample Size Justification

We conducted a series of simulation‐based power analyses to anticipate different outcomes for the primary hypotheses: H1 (comparing the frequency of prosocial versus antisocial behavior) and H2 (comparing the frequency of prosocial versus withdrawal behavior). Simulations were informed by previous findings from a similar Allocation game (Kip et al. 2025, Study 3) and were based on three sets of probabilities: Set 1 (prosocial = 55%, antisocial = 22.5%, withdrawal = 22.5%), Set 2 (prosocial = 50%, antisocial = 25%, withdrawal = 25%), and Set 3 (prosocial = 45%, antisocial = 27.5%, withdrawal = 27.5%). Based on these simulations, we aimed for a maximum sample size of *N* = 150 participants, which would provide 90% power to detect both effects of interest under Sets 1 and 2 (*α* = 0.05). To ensure sufficient statistical power in a feasible timeframe, we also determined that a minimum sample size of *N* = 110 participants would maintain 80% power to detect both effects (see power simulation OSF).

We were able to recruit a total of *N* = 98 participants. After exclusion, a final sample of *N* = 95 remained for the main analyses,^2^ ^,^ ^3^ which was slightly lower than our pre‐registered sample size based on a power analysis (*N* = 110).

### 2.2 Participants

The participants were recruited on campus of Tilburg University and through the social network of voluntary research student assistants from the Psychology bachelor's program. We recruited Caucasian^4^ and female^5^ participants (*M*_age_ = 20.28, SD_age_ = 1.88) as prior research suggests physiological differences, including those related to skin structure, blood flow, and hormonal influences, can vary across different genders (e.g., McFarland and Kadish 1991) and ethnic groups (e.g., Wesley and Maibach 2003). The decision to recruit only female participants aligns with the sample used by Paolini et al. (2016), allowing us to directly compare our findings with theirs. Additionally, we chose not to include gender as an independent variable due to practical considerations. Specifically, we anticipated that recruiting a sufficient number of male participants on campus during the project duration would not be feasible to ensure reliable gender comparisons, leading us to prioritize feasibility and alignment with prior research. The participants had no medical or psychiatric history and did not use any medication that could potentially interfere with the adrenergic or cardiovascular system. All participants were nonsmokers and did not use any recreational drugs^6^ 2 months prior to participation. Participants could only participate if they confirmed that they: were not in their ovulatory phase, did not perform physical exercise with a high or moderate intensity (> 6 h prior), did not drink alcohol or caffeinated beverages (> 6 h prior), were not actively sun tanning (> 2 h prior), and did not wear glasses. These criteria were based on prior work (Dreher et al. 2007; Jamal and Kamioka 2019; Kellogg Jr. 2006; Kelly et al. 2012; Kosonogov et al. 2017; Paolini et al. 2016; Sonkusare et al. 2019).

### 2.3 Design and Procedure

Data were collected during September 2022–May 2023 by the first author. Each participant individually took part in the study in a laboratory room at Tilburg University. Upon giving informed consent, the experimenter would set up the camera and provide make‐up remover to the participants if needed. Participants were then asked to sit up straight with their back against the backrest of the chair and to direct their face towards the computer screen at all times throughout the study. The participants were urged to remain in the same position as much as possible to prevent them from leaning over in their chair, which could result in invalid recordings (due to the camera would no longer being able to capture their entire face). The experimenter adjusted the camera to make sure that the participants' faces (and shoulders) were in the middle of the frame. The experimenter ensured that participants acclimatized to the environment (experimental room temperature: *M* = 19.16, ±0.70°C; humidity: *M* = 48.64, ±12.12) for approximately 10–15 min before starting the experiment (Fernández‐Cuevas et al. 2015; Van Beest and Sleegers 2019).

The experiment was administered using the software Inquisit 5 (Millisecond Software). Participants played two hypothetical Cyberball games (Williams and Jarvis 2006) in a fixed‐order within‐subjects design: inclusion followed by ostracism. This fixed sequence was chosen to align with Paolini et al. (2016) and fMRI studies on responses to ostracism (Gifuni et al. 2024; Krill and Platek 2009), which have consistently adopted this order to maximize the emotional impact of ostracism and ensure consistency in participant experiences. Unlike Paolini et al. (2016) who used a single baseline at the start of the session, we introduced a second baseline assessment, instructing participants to relax for 5 min prior to each hypothetical Cyberball game to obtain baseline measures of facial cutaneous temperature for the inclusion and ostracism conditions separately. In contrast to Paolini et al. (2016), where participants were led to believe they were interacting with two real players connected via intranet, participants were explicitly informed that they were playing against computer avatars. However, they were instructed to imagine these avatars as real players in a real‐life setting. Participants were told that the purpose of the game was not their ball‐tossing performance but rather to practice mental visualization. They were instructed to create a vivid mental picture of the game environment, imagining what the avatars might look like if they were real people, who these players might be, and where the game could be taking place. Each hypothetical Cyberball game consisted of three players (the participant and two computer avatars) and a total of 30 trials. A trial was defined as a single ball‐toss from one player to another. Participants always initiated the first ball‐toss of the game. During the inclusion condition, participants received every third ball‐toss throughout the game. In contrast, during the ostracism condition, participants did not receive any ball‐tosses for the entire game. After each hypothetical Cyberball game, we assessed participants' fundamental need satisfaction and subjective emotional responses related to the preceding game. The order of the questionnaires was counterbalanced across participants.

To examine different coping behaviors following ostracism, we used a hypothetical Allocation game (based on Leliveld et al. 2012; Kip et al. 2025, Study 3), which was administered after the assessment of psychological need satisfaction and emotions. The Allocation game was presented as a second mental visualization scenario. Participants were instructed to imagine that they were playing against one of the players from the second Cyberball game (i.e., the hypothetical ostracizer) and to base their responses on their experience during the second Cyberball game. Participants were endowed with 10 monetary units (each worth 10 cents), which they could allocate in one of three ways: (1) allocate units to increase the hypothetical earnings of the other player (i.e., prosocial behavior), (2) allocate units to decrease the hypothetical earnings of the other player (i.e., antisocial behavior), or (3) choose to do nothing (i.e., withdrawal). If participants chose to allocate, they could assign between a minimum of 1 and a maximum of 10 units. Each unit allocated to increase the other player's hypothetical earnings would result in a threefold increase (e.g., allocating 3 units would increase the other player's hypothetical earnings by 90 cents). Similarly, each unit allocated to decrease the other player's hypothetical earnings would result in a threefold reduction (e.g., allocating 3 units would decrease the other player's hypothetical earnings by 90 cents). Any remaining units would not be used and were hypothetically retained. To ensure that all responses had equal consequences for participants, they were explicitly told that their decisions and allocations would not influence their own earnings in any way. Participants were instructed to mentally visualize the entire scenario and imagine how they would respond to the other player in real life.

In the last part of the study, we asked participants how many ball‐tosses they thought they had received during each hypothetical Cyberball game [slider: 0%–100%]. In addition, we asked whether they could recall the consequences of their behavioral response during the hypothetical Allocation game to the other player [increased, decreased, or did nothing to the hypothetical earnings of the other player], and whether they had previous experience with Cyberball [yes, no, don't know, other namely…]. Finally, the participants reported their age and had the opportunity to write down their comments or feedback at the end of the study. All participants received a written debriefing and were given the opportunity to ask questions to the experimenter. Each participant was rewarded with €8 or course credit.

### 2.4 Measures

#### 2.4.1 Psychological Need Satisfaction

Psychological need satisfaction was assessed using 16 adapted items from the Need Satisfaction Scale (Van Beest and Williams 2006). The subscales (i.e., belonging, self‐esteem, control and meaningful existence) were presented in random order. All items were scored on a 7‐point rating (1 = not at all, 7 = very much), with higher scores indicating more need satisfaction. We computed average scores of the 16 items after inclusion (*α* = 0.90) and after ostracism (*α* = 0.82).

#### 2.4.2 Self‐Reported Emotional Responses

Self‐reported emotional responses were measured using 12 items. Six items from Çelik et al. (2013) were used for the subscales anger (angry, irritated, annoyed) and sadness (sad, down, lonely), while three items from Watson et al. (1988) assessed anxiety (anxious, tense, worried). Positive affect was measured with three items assessing happiness (cheerful, happy, optimistic). The subscales were presented in random order and rated on a 7‐point scale (1 = not at all, 7 = very much), with higher scores indicating stronger emotional experiences. Following reviewer recommendations, we deviated from the preregistration and included the assessment of negative and positive affect separately. Average scores were computed for negative affect (anger, sadness, and anxiety items) and positive affect (happiness items) separately after inclusion (*α* negative = 0.90, *α* positive = 0.92) and after ostracism (*α* negative = 0.90, *α* positive = 0.84).

#### 2.4.3 Behavior Type

Behavior type was coded based on participants' responses during the hypothetical Allocation game: antisocial (i.e., allocate units to decrease the hypothetical earnings of the other player), prosocial (i.e., allocate units to increase the hypothetical earnings of the other player), or withdrawal (i.e., do nothing—no impact on the hypothetical earnings of the other player).

### 2.5 Recording and Preparation of the Thermal Data

Facial cutaneous temperature variations were captured using a FLIR A655sc digital thermal camera (Flir Systems, Sweden). This camera has a detailed resolution with 640 × 480 pixels on its sensor, allowing it to detect very slight temperature differences as small as less than 30 mK (mK), ensuring precise measurements. For data recording, we used ResearchIR software.

To assess baseline levels of facial cutaneous temperature, we recorded the participants' facial cutaneous thermal responses during the 5‐min resting periods for the inclusion and ostracism conditions, respectively. As pre‐registered, 10 equally spaced frames were selected from the last 2 min of each baseline recording. With a sampling rate of 6.25 fps, baseline recordings consisted of 749–750 frames. To ensure even coverage across the 2‐min period, we selected one frame every 69 frames, starting from frame 0 (i.e., frames 68, 137, 207, 275, 344, 413, 482, 551, 620, and 689). This resulted in an 11.04‐s interval between selected frames and a total of 20 baseline thermal images per participant. Separate recordings were made for each ball toss during the two hypothetical Cyberball games resulting in a total of 60 thermal images per participant. As pre‐registered, we included thermal images for every third ball toss starting at trial 2 for the main analysis. We selected the final frame of each of these trials, as this is the point where participants would know with certainty whether they received the ball or not after it was tossed (i.e., the last frame of trial number: 2, 5, 8, 11, 14, 17, 20, 23, 26, and 29).

This resulted in 10 thermal images that were recorded during the inclusion condition and 10 thermal images that were recorded during the ostracism condition. In both conditions, the 10 selected images were from a trial during which the participant did not receive the ball. Changes in facial cutaneous temperature during the inclusion and the ostracism hypothetical Cyberball games were assessed as the temperature during the specific Cyberball condition minus the average temperature during the respective preceding baseline period. This means that positive scores indicated a relative increase in temperature, and negative scores indicated a relative decrease in temperature, compared to baseline.

#### 2.5.1 Regions of Interest (ROIs)

**FIGURE 1:**
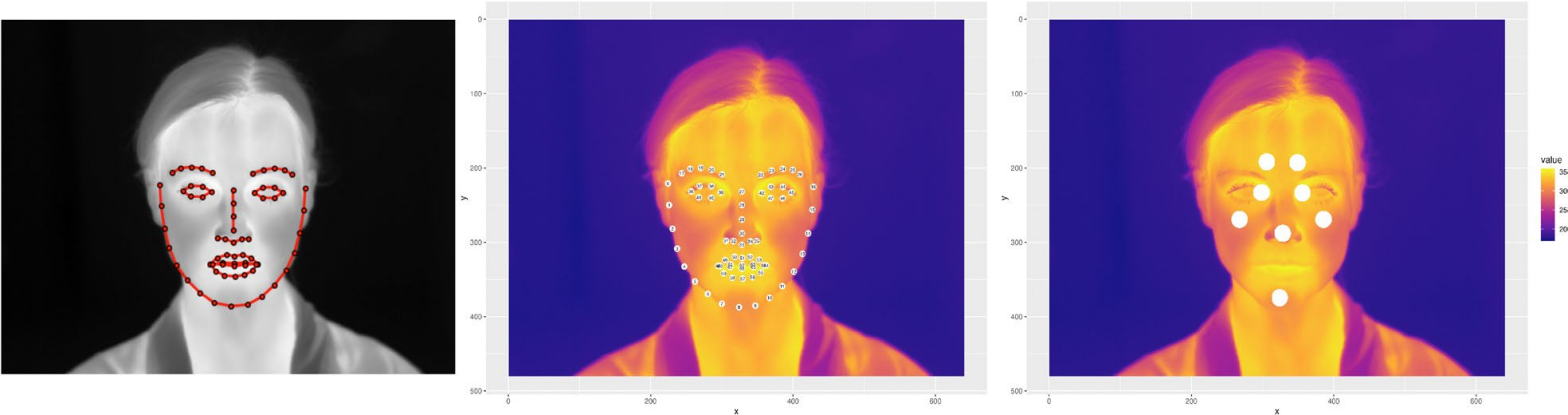
Facial landmark estimation on raw thermal image (left), numbered facial landmarks. Plotted on thermal image (middle), and eight valid regions of interest defined based on facial landmarks (right). *Note:* Thermal images are example images and are not included in the data analyses.

Temperature data were obtained for different ROIs of the face: nose tip, forehead left, forehead right, cheek left, cheek right, chin, left inner canthus, and right inner canthus (see Figure 1). The different ROIs were identified using facial landmarks obtained from each thermal image. These landmarks were detected using an algorithm developed by Kopaczka et al. (2016, 2017, 2018), which was trained on a facial thermal image database. This database includes manual landmark annotations and is freely available on GitHub for academic use (Kopaczka et al. 2016, 2017, 2018). The first author additionally performed a manual visual inspection to check whether the facial landmark detection and the located ROIs of the selected thermal images were valid (see Figure 1). If the ROIs for any of the selected thermal images were invalid (e.g., due to erroneous detection of the facial landmarks or movement of the head), the temperature value was registered as missing.^7^ Average temperatures per ROI were calculated based on the obtained temperatures of all pixels within the particular region (i.e., all pixels within a circle with a 10‐pixel radius).

#### 2.5.2 Included Thermal Data

Face detection was successful for 6678 (96.02%) out of the 6955 selected thermal images of the original 98 participants (prior to exclusion).^8^ Based on the manual visual inspection, ROI localization was valid for all ROIs in 4175 images (62.51%) (see Table 1). Among the other 2503 images, one or more ROIs were positioned incorrectly. After case‐wise exclusion of invalid ROI's, 6615 unique thermal images remained included. Additionally, 15 thermal images were excluded because participants (*n* = 13) were yawning or sighing, which distorted the temperature measurement of their face. The average temperatures for each ROI of the remaining 6600 images are reported in Table 1.

**TABLE 1:** Manual visual inspection of valid localization of different ROI's based on landmarks of the total amount thermal images for which a face was successfully detected (*k* = 6678) along with the *M* and SD temperature and number of participants (*N*) of the included images (*k* = 6600).

ROI	Valid cases (% of total cases)	*M* (SD) temperature (°C)	*N*
Nose	5794 (86.77%)	29.65 (3.78)	94
Chin	4710 (70.55%)	32.11 (1.15)	93
Left forehead	6114 (91.57%)	33.93 (0.62)	94
Right forehead	6163 (92.33%)	33.99 (0.66)	94
Left canthus	6325 (94.75%)	34.86 (0.54)	94
Right canthus	6376 (95.49%)	34.86 (0.49)	94
Left cheek	6067 (90.87%)	31.80 (1.13)	94
Right cheek	6231 (93.33%)	32.02 (1.13)	94

### 2.6 Planned Statistical Analyses

We planned to perform a multinomial logistic regression to test whether prosocial behavior was chosen more often than antisocial behavior following ostracism (H1) and to test whether prosocial behavior was chosen more often than withdrawal behavior following ostracism (H2), both tested against *α* < 0.05. In addition, we planned to perform linear mixed effects modeling to test whether participants showed bigger changes in facial cutaneous temperature (compared to baseline) when they were ostracized compared to included (H3) and whether the difference between ostracism and inclusion in facial cutaneous temperature changes increased over time (H4). Temperature change was predicted as a function of time, condition, and facial region of interest. Temperature change was mean‐centered. We planned to start with the maximum model and reduce the model complexity until the model converged using the step function from the lmerTest package (Kuznetsova et al. 2017). As pre‐registered, we deemed an effect statistically significant if the adjusted *p*‐value for multiple testing (*p*_FDR_) was lower than *α* = 0.01. Although we used a within‐subjects design, physiological data can still be noisy within participants due to factors like environmental influences or measurement error. To mitigate the risk of false positives and account for multiple comparisons, we applied this relatively conservative threshold to ensure that significant results were robust and not due to random fluctuations.

## 3 Results

Below we present our analyses on the full participant sample. We provide an additional analysis on a subsample in Supporting Information 1. This subsample excluded participants who indicated that they received ≤ 20% of the ball‐tosses during the inclusion condition (*n* = 2), and/or indicated that they received ≥ 26% of the ball‐tosses during the ostracism condition (*n* = 0), and/or did not recall the consequences of their behavioral response correctly (*n* = 0). The results of the subsample analysis generally remained consistent with those of the full sample.

### 3.1 Manipulation Checks Self‐Reported Measures

Four separate paired *t*‐tests (one‐sided) were conducted as manipulation checks to assess participants' experiences during the two Cyberball conditions based on the following self‐reported measures: The perceived percentage of received ball‐tosses was significantly lower following ostracism (*M* = 1.28, SD = 2.20) compared to inclusion (*M* = 35.74, SD = 10.31), *t*(94) = 31.76, *p* < 0.001, *d* = 3.26. Additionally, psychological need satisfaction was lower following ostracism (*M* = 1.86, SD = 0.64) compared to inclusion (*M* = 5.34, SD = 0.99), *t*(94) = 27.86, *p* < 0.001, *d* = 2.86. Negative affect was higher following ostracism (*M* = 4.32, SD = 1.30) compared to inclusion (*M* = 1.58, SD = 0.81), *t*(94) = −19.06, *p* < 0.001, *d* = −1.96. Positive affect was lower following ostracism (*M* = 1.67, SD = 0.78) compared to inclusion (*M* = 3.89, SD = 1.60), *t*(94) = 13.23, *p* < 0.001, *d* = 1.36. Overall, the manipulation checks based on these self‐reported measures were deemed successful.

### 3.2 Main Analyses

#### 3.2.1 Behavioral Responses After Ostracism

A total of 49 participants chose to withdraw (do nothing) during the hypothetical Allocation game, meaning they did not allocate any coins. In contrast, 29 participants allocated coins to respond antisocially (*M* = 3.75, SD = 2.03), and 17 participants allocated coins to respond prosocially (*M* = 4.47, SD = 3.45).

We tested whether prosocial responses were more frequent than antisocial (H1) and withdrawal (H2) responses. Multinomial logistic regression showed that more participants chose to respond antisocial (30.53%) compared to prosocial (17.89%) after experiencing ostracism. However, this difference was not significant, odds = 1.71, 95% CI [0.94, 3.10], *p* = 0.080. Participants were more likely to withdraw (51.58%) after ostracism than to act prosocial, odds = 2.88, 95% CI [1.66, 5.00], *p* < 0.001. Furthermore, participants showed a significant preference for withdrawal over antisocial responses, odds = 1.69, 95% CI [1.07, 2.67], *p* = 0.025. Thus, contrary to our hypotheses, withdrawal was the modal response rather than prosocial behavior.

#### 3.2.2 Facial Cutaneous Temperature Changes in Response to Ostracism

We tested whether participants showed bigger changes in facial cutaneous temperature (compared to respective baseline) during ostracism compared to inclusion (H3) and whether the difference between ostracism and inclusion in facial cutaneous temperature changes increased over time (H4). To ensure that any observed effects could not be attributed to environmental or temporal influences, we conducted additional exploratory analyses examining facial temperature stability during the two baseline periods (reported in Supporting Information 2).

**FIGURE 2:**
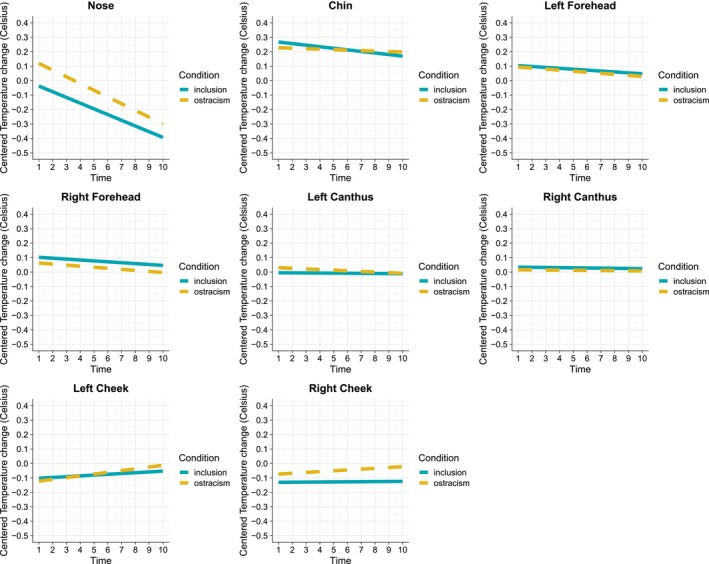
The fixed effects of time and condition on centered temperature change for each facial region of interest (ROI). *Note:* Plots derived from summarized data (i.e., not model estimates).

The maximal linear mixed‐effects model included fixed effects of time, condition, and facial region of interest (ROI) and all their interactions, the random intercepts for each subject, as well as the random slopes for the interaction between time and condition within each subject. Deviating from our preregistration, we excluded random slopes for ROI due to convergence issues when fitting the maximal model. The variable *time* refers to the 10 thermal images per condition, representing moments when participants did not receive the ball in chronological order. Fixed effects from the maximal model are presented in Table S3‐1 (Supporting Information 3). We used the stepwise() function from the lmerTest package (Kuznetsova et al. 2017) to reduce the model, but convergence issues remained (see Table S3‐2 in Supporting Information 3). Consequently, we deviated from our preregistration and simplified the random effects structure (Bates et al. 2015; Barr et al. 2013) by including only the random slopes for condition within subjects. ICC calculations supported the use of random effects, showing that 22% of the variance was due to between‐subject differences (ICC = 0.22), increasing to 36% when accounting for variation in response to condition (ICC = 0.36). The results of the final model are reported in Table 2 and illustrated in Figure 2, showing significant interaction effects between time and ROI (*p* < 0.001) and between condition and ROI (*p* < 0.001).

**TABLE 2:** Final model output: type III analysis of variance table for the fixed effects with Satterthwaite's method.

Fixed effects	SS	MS	*F*	df	*p*	*p*_FDR_
Time	5.35	5.35	34.98	1, 11,466.4	< 0.001**	< 0.001**
Condition	0.16	0.16	1.02	1, 90.5	0.316	0.316
ROI	30.37	4.34	28.38	7, 11,396.8	< 0.001**	< 0.001**
Time × ROI	20.60	2.94	19.25	7, 11,394.4	< 0.001**	< 0.001**
Condition ×ROI	7.22	1.03	6.75	7, 11,405.7	< 0.001**	< 0.001**

*Note:* *p*_FDR_ = false discovery rate correction applied to the seven *p*‐values for the fixed effects reported in this table. Significance of bold *p* values indicates significant values of ** *p* < 0.001.

##### 3.2.2.1 Interaction Effects Between Time and Different Facial ROIs

To decompose the omnibus interaction effect between Time and ROI reported in Table 2, we first assessed the effect of time on temperature change contrasting each facial region against another (see detailed statistics in Table S4‐1 in Supporting Information 4). There were significant interaction effects between time and ROI for the nose region compared to each other region (all *p*_FDR_'s < 0.001), meaning that temperature change over time was different for the nose region compared to all other regions. In addition, there were significant interaction effects between time and facial ROI for the left cheek compared to the chin region (*p*_FDR_ = 0.003), left forehead region (*p*_FDR_ = 0.007), and right forehead region (*p*_FDR_ = 0.007), meaning that temperature change over time was different for the left cheek compared to these three particular facial regions.

##### 3.2.2.2 Effects of Time Within Different Facial ROIs

To understand what was driving the interaction effects between time and different ROIs, we further explored the simple effects of time within each ROI (see Table 3, fixed effects of time). The effect of time was significant within the nose region (*b* = −0.044, *p*_FDR_ < 0.001) but not for any other ROI (all other *p*_FDR_'s > 0.024). This indicates that the interaction effects of time between the nose region and all other ROIs were driven by more pronounced temperature changes over time in the nose region. Notably, while the interaction effect between time and facial ROI was significant for the left cheek compared to the chin, left forehead, and right forehead, none of these regions showed significant simple effects of time (all *p*_FDR_'s > 0.055).

**TABLE 3:** Final model output: fixed effects of time and condition within each facial region of interest (ROI) with Satterthwaite's method.

	*b*	SE	df	*t*	*p*	*p*_FDR_
Fixed effects time						
Time (nose)	−0.044	0.004	11,404.383	−12.067	< 0.001**	< 0.001**
Time (chin)	−0.010	0.004	11,411.988	−2.465	0.014	0.055
Time (lhead)	−0.007	0.004	11,407.873	−2.013	0.044	0.101
Time (rhead)	−0.007	0.004	11,407.297	−2.035	0.042	0.101
Time (lcanthus)	−0.002	0.004	11,401.197	−0.678	0.498	0.725
Time (rcanthus)	−0.001	0.004	11,402.153	−0.324	0.746	0.796
Time (lcheek)	0.008	0.004	11,403.700	2.281	0.023	0.072
Time (rcheek)	0.003	0.004	11,403.416	0.803	0.422	0.676
Fixed effects condition						
Condition (nose)	0.124	0.030	271.196	4.082	< 0.001**	< 0.001**
Condition (chin)	0.013	0.032	350.233	0.390	0.697	0.796
Condition (lhead)	−0.011	0.030	255.029	−0.378	0.705	0.796
Condition (rhead)	−0.040	0.030	253.024	−1.344	0.180	0.361
Condition (lcanthus)	0.027	0.030	244.987	0.905	0.367	0.652
Condition (rcanthus)	−0.010	0.029	243.106	−0.352	0.725	0.796
Condition (lcheek)	0.005	0.030	257.383	0.159	0.874	0.874
Condition (rcheek)	0.079	0.030	250.484	2.676	0.008*	0.042

Random effects	Groups	Var	SD	*r*
Intercept	Subject	0.044	0.209	
Condition	Subject	0.041	0.202	−0.73
	Residual	0.153	0.391	

*Note:* Ref. category condition: inclusion, number of observations: 11,587, groups: subject, 94 *p*_FDR_ = false discovery rate correction was applied to the 16 *p*‐values reported in this table. Significance of bold *p* values indicates significant values of * *p* < 0.01,**p < 0.001.

##### 3.2.2.3 Interaction Effects Between Condition and Different Facial ROIs

To decompose the omnibus interaction effect between condition and ROI reported in Table 2, we first assessed the effect of condition on temperature change contrasting each facial region against another (see detailed statistics in Table S4‐2 in Supporting Information 4). There were significant interaction effects between condition and ROI for the nose region compared to all other regions (all *p*_FDR_'s < 0.002) except the right cheek (*p*_FDR_ = 0.225). This indicates that the difference in temperature change between conditions was different for the nose region compared to most other facial regions. In addition, there were significant interaction effects between condition and the right cheek region compared to the left forehead region (*p*_FDR_ = 0.005), right forehead region (*p*_FDR_ = < 0.001), and right canthus (*p*_FDR_ = 0.005), meaning that temperature change was different between conditions for the right cheek compared to these three particular facial regions.

##### 3.2.2.4 Effects of Condition Within Different Facial ROIs

To understand what was driving the interaction effects between condition and different ROIs, we further explored the simple effects of condition within each ROI (see Table 3, fixed effects of condition). Specifically, the temperature change differed significantly between the ostracism (*M* = −0.09, SE = 0.022, 95% CI [−0.14, −0.04]) and inclusion (*M* = −0.21, SE = 0.026, 95% CI [−0.27, −0.16]) conditions within the nose region (*p*_FDR_ < 0.001), but no significant differences were found for any of the other facial ROIs (all *p*_FDR_'s > 0.042). This suggests that the interaction effects between condition and ROI were driven by the temperature change difference observed in the nose region, rather than in the other facial regions. Crucially, the average nasal temperature change in both conditions showed a decrease compared to baseline. Moreover, and contrary to our hypothesis (H3), the average drop in nasal temperature was greater in the inclusion condition than in the ostracism condition. This suggests that, on average, ANS activity was stronger during the inclusion condition rather than the ostracism condition.

### 3.3 Exploratory Analyses

#### 3.3.1 Temperature Changes Nose Region

Since the nose region showed significant time and condition‐related differences in temperature changes, it stands out as the facial area where temperature variations were most pronounced. We therefore focused the following exploratory analyses exclusively on the nose region. This decision was based on the observed data and was not pre‐registered.

##### 3.3.1.1 Temperature Changes Nose Region in Relation to Behavioral Responses to Ostracism

We first explored whether the effects of time and condition on average temperature change in the nose region varied according to the participants' behavioral responses during the hypothetical Allocation game. A mixed‐effects model was performed to assess how temperature change was influenced by time, condition, and coping behavior and their interactions. In line with the main analyses, participants were treated as random intercepts to capture individual variability at the start of each condition, while condition was added as random slopes to account for how the effect of condition might vary across participants. To minimize the risk of Type I errors, we still used a conservative *α* level of 0.01, accounting for the inherent variability in physiological data. However, since the results were exploratory, we did not apply False Discovery Rate (FDR) correction. These results are reported in Table 4.

**TABLE 4:** Exploratory model output: type III analysis of variance table for the fixed effects of time, condition, and behavior type on nasal temperature change with Satterthwaite's method.

	SS	MS	*F*	df	*p*
Time	20.48	20.48	397.66	1, 1228.25	< 0.001**
Condition	0.08	0.08	1.63	1, 97.01	0.205
Behavior type	0.14	0.07	1.32	2, 97.28	0.273
Time × Condition	0.10	0.10	1.99	1, 1228.62	0.158
Time × Behavior type	0.08	0.04	0.81	2, 1228.40	0.444
Condition × Behavior type	0.02	0.01	0.18	2, 97.70	0.838
Time × Condition × Behavior type	0.78	0.39	7.59	2, 1228.75	< 0.001**

*Note:* Significance of bold *p* values indicates significant values of ** *p* < 0.001.

**FIGURE 3:**
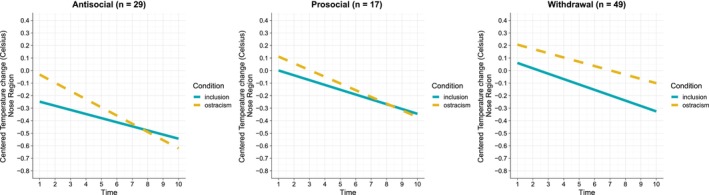
The fixed effects of time and condition on centered nasal temperature change for each type of behavioral response. *Note:* Plots derived from summarized data (i.e., not from model output).

The results showed a significant main effect of time (*p* < 0.001) and a significant three‐way interaction between time and condition, and behavior type on temperature change in the nose region (*p* < 0.001). The findings are shown in Figure 3. To further explore the significant three‐way interaction, we first explored the interaction effects of time and condition on temperature changes in the nose region between the different behavior groups. We then explored the main effects of condition and the interaction effects between time and condition within each behavior group.

##### 3.3.1.2 Effects of Time and Condition Between Behavior Groups

The interaction effects between time and condition were significantly different between antisocial respondents and those who chose to withdraw during the hypothetical Allocation game (*b* = 0.04, SE = 0.01, *t*(1231.02) = 3.85, *p* < 0.001). There was no significant difference between the interaction effects of time and condition of prosocial respondents compared to antisocial respondents (*b* = 0.02, SE = 0.01, *t*(1228.71) = 1.60, *p* = 0.109), nor of prosocial respondents compared to those who chose to withdraw during the hypothetical Allocation game (*b* = 0.02, SE = 0.01, *t*(1226.37) = 1.83, *p* = 0.067).

##### 3.3.1.3 Effects of Time and Condition Within Behavior Groups

There was no significant main effect of condition on average temperature change (inclusion: *M* = −0.38, SE = 0.17, ostracism: *M* = −0.29, SE = 0.14) for antisocial respondents (*b* = 0.23, SE = 0.19, *t*(103.74) = 1.22, *p* = 0.226). Crucially, the interaction effect between time and condition was significant for antisocial respondents (*b* = −0.03, SE = 0.01, *t*(1232.52) = −3.17, *p* = 0.002). Exploratory analyses of the simple effects of time on temperature change revealed that temperature changes decreased significantly during both the inclusion condition (*b* = −0.04, SE = 0.01, *t*(1233.11) = −5.79, *p* < 0.001) and the ostracism condition (*b* = −0.06, SE = 0.01, *t*(1230.56) = −10.44, *p* < 0.001). In both conditions, average temperature changes reflected a decrease compared to baseline, showing greater decreases over time, with the effect being more pronounced during ostracism compared to inclusion (see Figure 3). These findings suggest that antisocial respondents during the hypothetical Allocation game were more physiologically reactive during ostracism relative to inclusion.

There was no significant main effect of condition on average temperature change (inclusion: *M* = −0.17, SE = 0.22, ostracism: *M* = −0.12, SE = 0.16) for prosocial respondents (*b* = 0.08, 0.22, *t*(91.61) = 0.37, *p* = 0.709). In addition, there was no significant interaction between time and condition for prosocial respondents [*b* = −0.01, SE = 0.01, *t*(1225.50) = −0.73, *p* = 0.468].

There was no significant main effect of condition on average temperature change (inclusion: *M* = −0.13, SE = 0.13, ostracism: *M* = 0.04, SE = 0.10) for those who chose to withdraw (*b* = 0.10, SE = 0.14, *t*(99.46) = 0.72, *p* = 0.472). In addition, there was no significant interaction between time and condition for those who chose withdrawal (*b* = −0.01, SE = 0.01, *t*(1228.27) = −2.20, *p* = 0.028). Notably, those who chose to withdraw showed a temperature increase relative to baseline from Time 1 to Time 6 during ostracism. And although the overall slope was negative, indicating a numerical decrease over time, this was the only condition in which the average temperature across all time points increased relative to baseline. This pattern did not occur in any other behavioral group (prosocial or antisocial coping) or in the inclusion conditions, making it uniquely characteristic of those who chose to withdraw during the hypothetical Allocation game (see Figure 3).

##### 3.3.1.4 Temperature Changes Nose Region in Relation to Self‐Reported Responses to Ostracism

For exploratory purposes, we report the correlations, means, and standard deviations of self‐reported psychological need satisfaction, negative affect, positive affect, and nasal temperature changes in Table 5. Notably, nasal temperature change during inclusion (T1) was significantly correlated with nasal temperature change during ostracism (T2) (*r*(74) = 0.42, *p* < 0.001). However, nasal temperature change during either condition did not significantly correlate with any of the self‐reported measures, including psychological need satisfaction, negative affect, and positive affect.

**TABLE 5:** Pearson correlations, means, and standard deviations of self‐reported psychological need satisfaction, positive affect, and negative affect measured after inclusion (T1) and ostracism (T2), alongside centered nasal temperature change recorded during inclusion (T1) and ostracism (T2).

	1	2	3	4	5	6	7	8
1. Need Satisfaction (T1)	—	−0.07	−0.50**	0.18	0.39**	0.10	0.08	−0.11
2. Need satisfaction (T2)		—	0.07	−0.61**	−0.06	0.49**	0.06	0.10
3. Negative affect (T1)			—	0.19	−0.13	−0.04	0.05	0.29
4. Negative affect (T2)				—	0.25	−0.34	−0.07	−0.09
5. Positive affect (T1)					—	0.24	0.04	0.04
6. Positive affect (T2)						—	0.05	0.15
7. Temperature change (T1)							—	0.42**
8. Temperature change (T2)								—
*M* (SD)	5.36 (0.95)	1.85 (0.65)	1.55 (0.69)	4.32 (1.31)	3.87 (1.59)	1.68 (0.78)	−0.21 (0.89)	−0.08 (0.67)

*Note:* Correlations for temperature change (T1 and T2) are based on the average of 10 frames within each condition for each participant.

** *p* < 0.01, *p*‐values adjusted for multiple testing.

## 4 Discussion

Ostracism triggers psychophysiological responses associated with distress. We investigated different coping behaviors following ostracism and explored whether these were preceded by unique facial thermal signatures. Contrary to previous findings by Kip et al. (2025), the majority of participants opted to withdraw after ostracism, followed by antisocial and prosocial coping. Thermal infrared imaging was used to detect facial cutaneous temperature responses as a proxy for ANS activity during ostracism. While we investigated facial temperature responses across several regions of interest, we observed considerable variability only in the nose region. Moreover, both the inclusion and ostracism conditions were associated with a decrease in nasal temperature compared to baseline, but this drop was greater during inclusion rather than during ostracism. Contrary to previous research (Mazzone et al. 2017; Paolini et al. 2016; Ponsi et al. 2019), this finding suggests that ANS activity was stronger during inclusion compared to ostracism. Moreover, we found no evidence that the observed difference in nasal temperature change between the conditions increased over time, as hypothesized. Crucially, exploratory findings suggested that antisocial respondents exhibited distinct nasal thermal patterns when compared to those who chose to withdraw after ostracism. Specifically, antisocial respondents appeared more physiologically reactive, as indicated by steeper nasal temperature decreases during ostracism relative to inclusion. This provides a first hint that coping responses after ostracism may be associated with distinct nasal temperature patterns, warranting further investigation.

### 4.1 Coping Behaviors Following Ostracism

Literature on ostracism typically categorizes coping behaviors in response to ostracism into antisocial, prosocial, and withdrawal responses (Ren et al. 2018; Smart Richman and Leary 2009; Williams 2009). We observed the occurrence of these three different coping behaviors following ostracism during a hypothetical Allocation game (based on Leliveld et al. 2012). In contrast to Kip et al. (2025, Study 3), in which prosocial responses were most common, the current study showed that withdrawal was the main reaction after ostracism. This difference may be attributed to differences in experimental design. Kip et al. used a between‐subjects design, meaning participants could not compare or adjust their responses based on a prior experience. In contrast, the current study employed a within‐subjects design, where participants first experienced inclusion before ostracism. First, because of their initial experience of inclusion, participants may have perceived the subsequent experience of ostracism as more unexpected and thus more severe by comparison. Second, although there was no explicit assessment following inclusion, participants may have mentally compared how they would have responded after inclusion when deciding how to respond after ostracism. This internal comparison, only possible in a within‐subjects design, could have influenced their behavior, potentially leading them to adjust their response, for instance, choosing to be less prosocial or not prosocial at all after ostracism.

### 4.2 Nasal Cutaneous Temperature Changes During Ostracism

While the manipulation of ostracism (vs. inclusion) was successful in terms of self‐reported measures indicating reduced psychological need satisfaction, heightened negative affect, and reduced positive affect, the observed temperature changes in the nose region were misaligned. In contrast to prior findings (Mazzone et al. 2017; Paolini et al. 2016; Ponsi et al. 2019), our findings did not support the notion that ostracism is associated with increased ANS activation. Instead, a stronger nasal temperature drop was observed during inclusion rather than ostracism. Moreover, exploratory analyses did not provide evidence that self‐reported measures and nasal cutaneous temperature changes were correlated. This relates to the notion of response coherence (e.g., Ekman 1992; Lazarus 1991; Levenson 1994), showing that different emotional response systems (experiential, behavioral, physiological) do not always align perfectly (Brown et al. 2020; Mauss et al. 2005). Alternatively, the observed discrepancy between self‐reported and thermal responses suggests that the current hypothetical Cyberball games, using mental imagery rather than deception or real interactions, do not sufficiently provoke ANS activity detectable through facial temperature changes. Stronger emotional triggers may be required to elicit more pronounced ANS activation (Cacioppo et al. 2000). Speaking to this, the Cyberball paradigm's ecological validity has been questioned, as its abstract and minimalistic nature diminishes psychological distress compared to real‐life interactions (e.g., Eisenberger 2015b; Parsons 2015). This calls for ecologically valid stimulus presentation and more immersive virtual environments to better simulate experiences of ostracism and provoke stronger emotional responses (Kassner et al. 2012; Wirth et al. 2011; Meral et al. 2022).

### 4.3 Nasal Temperature Signatures of Coping Behaviors

Our exploratory findings revealed that only antisocial respondents showed a more pronounced decrease in nasal temperature during the preceding ostracism experience relative to inclusion. This pronounced decrease suggests a strong affective response to ostracism, likely driven by heightened SNS activity, which is associated with active coping behaviors. Specifically, a drop in nasal temperature is commonly observed in response to stress, reflecting SNS activation. This observation aligns with previous research showing that a drop in nasal temperature can indicate increased arousal or negative emotional states, both in nonhuman animals (Diaz‐Piedra et al. 2019; Ioannou et al. 2015; Kuraoka and Nakamura 2011; Nakayama et al. 2005) and humans (Or and Duffy 2007; Ioannou et al. 2013; Naemura et al. 1993). This heightened reactivity among antisocial respondents may reflect stronger negative affect during ostracism, or alternatively, individuals with more aggressive tendencies may react more strongly to ostracism. Importantly, the physiological responses observed may not only result from these tendencies and emotional states, but could also contribute to the development or reinforcement of antisocial traits themselves (Cacioppo et al. 2000).

In contrast, participants who chose to withdraw (i.e., did nothing) after ostracism showed an increase in nasal temperature during ostracism compared to baseline, which may reflect lower SNS activation or a shift to parasympathetic nervous system (PNS) involvement (Asano et al. 2010; Ioannou et al. 2014; Kim et al. 2018; Magnon et al. 2021; Panasiti et al. 2016; Nozawa and Tacano 2009; Wehrwein et al. 2016). This pattern seems consistent with social avoidance tendencies, as studied by Duijndam et al. (2020), who found that socially inhibited individuals already had lower baseline parasympathetic activity at rest, and as a result, the expected reduction in parasympathetic activity during emotional stress did not happen as strongly as it typically would. Moreover, withdrawal in our study was characterized by disengagement or inaction rather than active avoidance, which may be more closely linked to relaxation, stress‐free states, and positive emotional processing, as indicated by increased nasal temperature (Esposito et al. 2015; Genno et al. 1997; Kaushik et al. 2006; Salazar‐López et al. 2015).

Based on the above, temperature changes might reflect emotional valence, with nasal temperature decreases typically linked to negative emotions and increases to positive ones. However, the observed nasal temperature changes during the inclusion condition challenge this interpretation. We observed a decrease in nasal temperature compared to baseline during inclusion, which further decreased over time. This raises the question of whether inclusion in our study was associated with negative emotional states. However, based on self‐report measures, we found that inclusion was linked to relatively stronger positive affect and weaker negative affect compared to ostracism. That said, we cannot rule out the possibility that participants experienced more negative affect and less positive affect during inclusion compared to their emotional state before the experiment. Since we did not obtain a baseline measure prior to both conditions, it remains unclear how participants' affect during inclusion compared to their initial state. Alternatively, the direction of temperature changes might reflect cognitive engagement or information processing (e.g., Cardone et al. 2022; Kang et al. 2006). In this case, the decrease could signal mental effort or task involvement (Abdelrahman et al. 2017; Veltman and Vos 2005) rather than a negative emotional response. Conversely, temperature increases may indicate disengagement or low cognitive load (Panasiti et al. 2019), particularly among those who withdrew. This is especially relevant because, during inclusion, participants actively engaged by clicking the mouse to throw the ball to others, whereas during ostracism, they remained passive observers without any interaction.

Moreover, our exploratory findings did not show evidence that nasal temperature changes during inclusion or ostracism were associated with self‐reported positive or negative affect. This suggests that the direction of temperature changes may not be a straightforward indicator of emotional valence but could also reflect cognitive processes or task involvement. One possible explanation for the lack of a significant correlation between nasal temperature changes and self‐reported affect is that the physiological responses observed were not strong enough to produce a clear association with subjective experience. Thermal responses can be subtle and influenced by multiple factors, and self‐reported emotions may not always align perfectly with physiological markers due to differences in timing, individual variability, or introspective accuracy (Ciuk et al. 2015; Kassam and Mendes 2013). In particular, Kassam and Mendes (2013) demonstrated that the act of reporting on emotional states can itself alter physiological responses, suggesting that measurement effects may contribute to discrepancies between self‐reported affect and physiological markers. This divergence aligns with Damasio's (2004) distinction between bioregulatory reactions (emotions) and the conscious interpretation of those reactions (feelings). In sum, temporal and conceptual distinctions may explain why thermal responses and self‐reported affect do not always correspond in a straightforward manner. Alternatively, temperature changes may capture a mix of affective and cognitive processes, making it difficult to isolate their emotional significance in relation to self‐reported measures.

### 4.4 Future Directions

While we assumed that experiences of inclusion and ostracism would intensify gradually throughout the Cyberball paradigm, one could alternatively expect an initial stress‐related temperature change followed by a return to baseline due to habituation or ongoing emotion regulation processes (e.g., Oken et al. 2015; Dhabhar 2018). However, we did not observe such adaptation within 30 ball‐tosses. Instead, nasal temperature continued to decrease, suggesting a prolonged physiological response. This aligns with Kelly et al. (2012), who found no evidence of habituation in skin conductance during ostracism. However, unlike their findings of habituation in SCL during inclusion, we did not observe this in facial temperature, possibly due to differences in autonomic processes (SCL reflects more rapid sympathetic responses, whereas facial temperature is influenced by slower vasomotor regulation). If physiological adaptation in nasal thermal responses requires more time, prolonged experiences of ostracism and inclusion (e.g., 90 ball‐tosses) could help determine whether habituation eventually occurs. Additionally, as cognitive and emotion regulation can shape physiological stress responses (de Veld et al. 2012; Yuan et al. 2015; Jentsch and Wolf 2020), future studies could manipulate strategies like cognitive reappraisal or suppression to assess their impact on thermal adaptation in relation to different coping mechanisms.

Despite the widespread use of the Cyberball paradigm in social neuroscience to investigate responses to ostracism, our findings using hypothetical Cyberball games cast doubt on its effectiveness in eliciting physiological responses of distress that are detectable through thermal infrared imaging. While the Cyberball paradigm facilitates comparisons across studies using measures such as skin conductance level (e.g., Kelly et al. 2012; Kouchaki and Wareham 2015; Sijtsema et al. 2011), fMRI (e.g., Eisenberger 2012, 2015a; Rotge et al. 2015), and heart rate deceleration (e.g., Gunther Moor et al. 2010), our study suggests the need for additional experimental methods to accurately assess distress‐related temperature changes. By comparing temperature changes in different body regions, such as the nose versus extremities like the fingertips, researchers can evaluate whether changes are localized to specific areas typically associated with SNS activity. As suggested by Kosonogov et al. (2017), integrating thermal imaging with other physiological measures, such as heart rate variability or galvanic skin responses, could offer further insights into ANS activity, helping distinguish psychological distress from environmental influences and other intraindividual changes, such as cognitive effort. Monitoring these responses alongside stronger manipulations of ostracism may clarify the underlying causes of observed temperature changes and clarify the relationship between self‐reported and physiological measures. Thermal imaging, with its non‐invasive and contact‐free nature, provides a practical tool for real‐time monitoring of these responses during social interactions, offering a less intrusive alternative to traditional physiological measurement techniques.

### 4.5 Strengths and Limitations

A key strength of the study is that we pre‐registered our data processing plan and analyses, using (semi)automated methods for selecting facial ROIs and temperature data. The study benefits from a relatively large sample size, though future research should use even larger samples to enable more robust comparisons of nasal temperature signatures based on different behavioral responses following ostracism. In line with established protocols (e.g., Paolini et al. 2016; Krill and Platek 2009), we employed a fixed order of conditions, where inclusion was always presented before ostracism. Although this sequence was chosen to maximize the emotional impact of ostracism and to avoid confounding effects of inclusion being perceived as unexpectedly rewarding, it does not allow us to completely disentangle condition effects from potential order effects of the experimental conditions. To mitigate this limitation, we introduced an additional baseline assessment before the ostracism condition and calculated temperature changes during each experimental condition relative to their respective baseline. Nonetheless, future research could explore counterbalanced or randomized designs to further validate the robustness of the findings. Finally, our sample consisted exclusively of women, which aligns with previous research by Paolini et al. (2016) but limits the generalizability of our results. Gender‐specific physiological and behavioral responses in relation to ostracism should be examined in future studies to expand the applicability of these findings across diverse populations.

## 5 Conclusion

This study highlights different coping behaviors following ostracism and offers exploratory evidence that they may be preceded by distinct nasal temperature signatures. Withdrawal was the most common response after ostracism, followed by antisocial and prosocial behavior. Although no overall increase in ANS activity was observed during ostracism, based on average nasal temperature changes, exploratory analyses revealed a notable distinction across coping strategies. Only participants who responded antisocially showed steeper nasal temperature decreases during ostracism compared to inclusion, suggesting stronger physiological reactivity, particularly relative to those who withdrew. Future research should combine thermal imaging with additional physiological measures and use stronger ostracism manipulations to better understand the relationship between facial thermal responses and coping behaviors in the context of social exclusion.

## Author Contributions

**Anneloes Kip:** conceptualization, formal analysis, investigation, methodology, project administration, resources, visualization, writing – original draft, writing – review and editing. **Thorsten M. Erle:** conceptualization, methodology, supervision, writing – review and editing. **Ilja van Beest:** conceptualization, funding acquisition, methodology, supervision, writing – review and editing.

## Ethics Statement

The study was approved by the Ethics Review Board of the School of Social and Behavioral Sciences at Tilburg University (TSB_RP344).

## Conflicts of Interest

The authors declare no conflicts of interest.

## Supporting information


Data S1.

Data S2.

Data S3.

Data S4.

## Data Availability

The preregistration, anonymized data, code, power simulation, materials, and supporting information for this research are available at the Open Science Framework (OSF): https://osf.io/mhc67/.

## Endnotes

^1^
Our hypothesis about coping deviated from the preregistration. In the preregistration we stated that we would compare coping also between inclusion and ostracism condition. However, we did not provide participants an opportunity to cope with inclusion and we had forgotten to update this in our preregistration. Consequently, our analysis of behavioral responses are only provided for the ostracism experience.

^2^
We deviated from the preregistration and extended the data collection in an attempt to reach the desired sample size of *N* = 110.

^3^

*n* = 1 Participant accidentally participated twice in our study. We only included the first response in the main analyses reported in this paper.

^4^

*n* = 1 Participant had Asian ethnicity and participated for educational purposes. In personal agreement her data was excluded from data‐analyses for the current paper.

^5^

*n* = 1 Participant was AFAB (assigned female at birth) and identified as male.

^6^
To stimulate the recruitment of participants, we deviated from the preregistration and lowered the requirement regarding recreational drug‐use from 1 year to a minimal period of > 2 months prior to participation.

^7^
We deviated from the preregistration and coded temperature values as missing (invalid) without checking whether the preceding image was valid instead. This decision was made because we realized that any issue causing a temperature value to be coded as missing (e.g., head turning, hand in face) was highly likely to affect the preceding image as well.

^8^
Due to technical malfunctions with the camera, we were unable to acquire all 80 recordings (20 baseline recordings and 60 ball‐toss recordings) for 16 participants. For one additional participant, we were unable to record the experiment at all.

## References

[psyp70081-bib-0001] Abdelrahman, Y. , E. Velloso , T. Dingler , A. Schmidt , and F. Vetere . 2017. “Cognitive Heat: Exploring the Usage of Thermal Imaging to Unobtrusively Estimate Cognitive Load.” Proceedings of the ACM on Interactive, Mobile, Wearable and Ubiquitous Technologies 1, no. 3: 1–20. 10.1145/3130898.

[psyp70081-bib-0002] Asano, H. , H. Onogaki , T. Muto , S. Yokoyama , and H. Ide . 2010. “Stress Presumption of the Long Driving Using the Facial Thermal Image.” Journal of Robotics and Mechatronics 22, no. 6: 751–757. 10.20965/jrm.2010.p0751.

[psyp70081-bib-0003] Axelrod, R. 1980. “More Effective Choice in the Prisoner's Dilemma.” Journal of Conflict Resolution 24, no. 3: 379–403. 10.1177/002200278002400301.

[psyp70081-bib-0004] Barr, D. J. , R. Levy , C. Scheepers , and H. J. Tily . 2013. “Random Effects Structure for Confirmatory Hypothesis Testing: Keep It Maximal.” Journal of Memory and Language 68, no. 3: 255–278. 10.1016/j.jml.2012.11.001. PMC3881361 24403724

[psyp70081-bib-0005] Bates, D. , R. Kliegl , S. Vasishth , and H. Baayen . 2015. “Parsimonious Mixed Models.” 10.48550/arXiv.1506.04967.

[psyp70081-bib-0006] Baumeister, R. F. , and M. R. Leary . 1995. “The Need to Belong: Desire for Interpersonal Attachments as a Fundamental Human Motivation.” Psychological Bulletin 117, no. 3: 497–529. 10.1037/0033-2909.117.3.497. 7777651

[psyp70081-bib-0007] Baumeister, R. F. , and K. D. Vohs . 2004. “Four Roots of Evil.” In The Social Psychology of Good and Evil, edited by A. G. Miller , 85–101. Guilford Press.

[psyp70081-bib-0008] Beauchaine, T. 2001. “Vagal Tone, Development, and Gray's Motivational Theory: Toward an Integrated Model of Autonomic Nervous System Functioning in Psychopathology.” Development and Psychopathology 13, no. 2: 183–214. 10.1017/S0954579401002012. 11393643

[psyp70081-bib-0009] Bentsianov, B. , and A. Blitzer . 2004. “Facial Anatomy.” Clinics in Dermatology 22, no. 1: 3–13. 10.1016/j.clindermatol.2003.11.011. 15158538

[psyp70081-bib-0012] Brown, C. L. , N. Van Doren , B. Q. Ford , I. B. Mauss , J. W. Sze , and R. W. Levenson . 2020. “Coherence Between Subjective Experience and Physiology in Emotion: Individual Differences and Implications for Well‐Being.” Emotion 20, no. 5: 818–829. 10.1037/emo0000579. 30869944 PMC8158438

[psyp70081-bib-0014] Cacioppo, J. T. , L. G. Tassinary , and G. G. Berntson . 2000. “Psychophysiological Science: Interdisciplinary Approaches to Classic Questions About the Mind.” In Handbook of Psychophysiology, edited by J. T. Cacioppo , L. G. Tassinary , and G. G. Berntson , 1–16. Cambridge University Press.

[psyp70081-bib-0015] Cannon, W. B. 1915. Bodily Changes in Pain, Hunger, Fear, and Rage. D. Appleton and Company.

[psyp70081-bib-0016] Cardone, D. , and A. Merla . 2017. “New Frontiers for Applications of Thermal Infrared Imaging Devices: Computational Psychopshysiology in the Neurosciences.” Sensors 17, no. 5: 1042. 10.3390/s17051042. 28475155 PMC5469647

[psyp70081-bib-0017] Cardone, D. , D. Perpetuini , C. Filippini , et al. 2022. “Classification of Drivers' Mental Workload Levels: Comparison of Machine Learning Methods Based on ECG and Infrared Thermal Signals.” Sensors 22, no. 19: 7300. 10.3390/s22197300. 36236399 PMC9572767

[psyp70081-bib-0018] Çelik, P. , J. Lammers , I. van Beest , M. H. Bekker , and R. Vonk . 2013. “Not All Rejections Are Alike; Competence and Warmth as a Fundamental Distinction in Social Rejection.” Journal of Experimental Social Psychology 49, no. 4: 635–642. 10.1016/j.jesp.2013.02.010.

[psyp70081-bib-0019] Chester, D. S. , N. I. Eisenberger , R. S. Pond Jr. , S. B. Richman , B. J. Bushman , and C. N. DeWall . 2014. “The Interactive Effect of Social Pain and Executive Functioning on Aggression: An fMRI Experiment.” Social Cognitive and Affective Neuroscience 9, no. 5: 699–704. 10.1093/scan/nst038. 23482622 PMC4014110

[psyp70081-bib-0020] Chester, D. S. , D. R. Lynam , R. Milich , and C. N. DeWall . 2018. “Neural Mechanisms of the Rejection–Aggression Link.” Social Cognitive and Affective Neuroscience 13, no. 5: 501–512. 10.1093/scan/nsy025. 29618118 PMC6007431

[psyp70081-bib-0021] Cho, Y. , N. Bianchi‐Berthouze , M. Oliveira , C. Holloway , and S. Julier . 2019. “Nose Heat: Exploring Stress‐Induced Nasal Thermal Variability Through Mobile Thermal Imaging.” In International Conference on Affective Computing and Intelligent Interaction, 566–572. IEEE. 10.1109/ACII.2019.8925453.

[psyp70081-bib-0022] Ciuk, D. J. , A. S. Troy , and M. C. Jones . 2015. “Measuring Emotion: Self‐Reports vs. Physiological Indicators [Preprint].” SSRN. 10.2139/ssrn.2595359.

[psyp70081-bib-0023] Cruz‐Albarran, I. A. , J. P. Benitez‐Rangel , R. A. Osornio‐Rios , and L. A. Morales‐Hernandez . 2017. “Human Emotions Detection Based on a Smart‐Thermal System of Thermographic Images.” Infrared Physics & Technology 81: 250–261. 10.1016/j.infrared.2017.01.002.

[psyp70081-bib-0024] Damasio, A. R. 2004. “Emotions and Feelings. A Neurobiological Perspective.” In Feelings and Emotions: The Amsterdam Symposium, edited by A. S. R. Manstead , N. Frijda , and A. Fischer , 49–57. Cambridge University Press. 10.1017/CBO9780511806582.004.

[psyp70081-bib-0025] de Veld, D. M. , J. M. Riksen‐Walraven , and C. de Weerth . 2012. “The Relation Between Emotion Regulation Strategies and Physiological Stress Responses in Middle Childhood.” Psychoneuroendocrinology 37, no. 8: 1309–1319. 10.1016/j.psyneuen.2012.01.004. 22309825

[psyp70081-bib-0026] Derakhshan, A. , M. Mikaeili , A. M. Nasrabadi , and T. Gedeon . 2019. “Network Physiology of ‘Fight or Flight’ Response in Facial Superficial Blood Vessels.” Physiological Measurement 40, no. 1: 014002. 10.1088/1361-6579/aaf089. 30523843

[psyp70081-bib-0027] DeWall, C. N. , and S. B. Richman . 2011. “Social Exclusion and the Desire to Reconnect.” Social and Personality Psychology Compass 5, no. 11: 919–932. 10.1111/j.1751-9004.2011.00383.x.

[psyp70081-bib-0028] Dhabhar, F. S. 2018. “The Short‐Term Stress Response: Mother Nature's Mechanism for Enhancing Protection and Performance Under Conditions of Threat, Challenge, and Opportunity.” Frontiers in Neuroendocrinology 49: 175–192. 10.1016/j.yfrne.2018.03.004. 29596867 PMC5964013

[psyp70081-bib-0029] Diaz‐Piedra, C. , E. Gomez‐Milan , and L. L. Di Stasi . 2019. “Nasal Skin Temperature Reveals Changes in Arousal Levels due to Time on Task: An Experimental Thermal Infrared Imaging Study.” Applied Ergonomics 81: 102870. 10.1016/j.apergo.2019.06.001. 31422278

[psyp70081-bib-0030] Dreher, J. C. , P. J. Schmidt , P. Kohn , D. Furman , D. Rubinow , and K. F. Berman . 2007. “Menstrual Cycle Phase Modulates Reward‐Related Neural Function in Women.” Proceedings of the National Academy of Sciences 104, no. 7: 2465–2470. 10.1073/pnas.0605569104. PMC1892961 17267613

[psyp70081-bib-0031] Drummond, P. D. , L. Camacho , N. Formentin , T. D. Heffernan , F. Williams , and T. E. Zekas . 2003. “The Impact of Verbal Feedback About Blushing on Social Discomfort and Facial Blood Flow During Embarrassing Tasks.” Behaviour Research and Therapy 41, no. 4: 413–425. 10.1016/S0005-7967(02)00021-9. 12643965

[psyp70081-bib-0032] Duijndam, S. , A. Karreman , J. Denollet , and N. Kupper . 2020. “Emotion Regulation in Social Interaction: Physiological and Emotional Responses Associated With Social Inhibition.” International Journal of Psychophysiology 158: 62–72. 10.1016/j.ijpsycho.2020.09.013. 33086100

[psyp70081-bib-0127] Eisenberger, N. I. 2013. “Social Ties and Health: A Social Neuroscience Perspective.” Current Opinion in Neurobiology 23, no. 3: 407–413. 10.1016/j.conb.2013.01.006. 23395461 PMC3664098

[psyp70081-bib-0033] Eisenberger, N. I. 2012. “The Pain of Social Disconnection: Examining the Shared Neural Underpinnings of Physical and Social Pain.” Nature Reviews Neuroscience 13, no. 6: 421–434. 10.1038/nrn3231. 22551663

[psyp70081-bib-0034] Eisenberger, N. I. 2015a. “Meta‐Analytic Evidence for the Role of the Anterior Cingulate Cortex in Social Pain.” Social Cognitive and Affective Neuroscience 10, no. 1: 1–2. 10.1093/scan/nsu120. 25210052 PMC4994854

[psyp70081-bib-0035] Eisenberger, N. I. 2015b. “Social Pain and the Brain: Controversies, Questions, and Where to Go From Here.” Annual Review of Psychology 66: 601–629. 10.1146/annurev-psych-010213-115146. 25251482

[psyp70081-bib-0036] Eisenberger, N. I. , S. L. Gable , and M. D. Lieberman . 2007. “Functional Magnetic Resonance Imaging Responses Relate to Differences in Real‐World Social Experience.” Emotion 7, no. 4: 745–754. 10.1037/1528-3542.7.4.745. 18039043

[psyp70081-bib-0037] Eisenberger, N. I. , and M. D. Lieberman . 2004. “Why Rejection Hurts: A Common Neural Alarm System for Physical and Social Pain.” Trends in Cognitive Sciences 8, no. 7: 294–300. 10.1016/j.tics.2004.05.010. 15242688

[psyp70081-bib-0038] Ekman, P. 1992. “An Argument for Basic Emotions.” Cognition & Emotion 6, no. 3–4: 169–200. 10.1080/02699939208411068.

[psyp70081-bib-0039] Esposito, G. , J. Nakazawa , S. Ogawa , R. Stival , D. L. Putnick , and M. H. Bornstein . 2015. “Using Infrared Thermography to Assess Emotional Responses to Infants.” Early Child Development and Care 185, no. 3: 438–447. 10.1080/03004430.2014.932153. 29527089 PMC5844285

[psyp70081-bib-0040] Fernández‐Cuevas, I. , J. C. B. Marins , J. A. Lastras , et al. 2015. “Classification of Factors Influencing the Use of Infrared Thermography in Humans: A Review.” Infrared Physics & Technology 71: 28–55. 10.1016/j.infrared.2015.02.007.

[psyp70081-bib-0041] Ferris, L. J. 2019. “Hurt Feelings: Physical Pain, Social Exclusion, and the Psychology of Pain Overlap.” In Current Directions in Ostracism, Social Exclusion and Rejection Research, edited by S. C. Rudert , R. Greifeneder , and K. D. Williams , 100–119. Routledge. 10.4324/9781351255912.

[psyp70081-bib-0043] Genno, H. , K. Ishikawa , O. Kanbara , et al. 1997. “Using Facial Skin Temperature to Objectively Evaluate Sensations.” International Journal of Industrial Ergonomics 19, no. 2: 161–171. 10.1016/S0169-8141(96)00011-X.

[psyp70081-bib-0045] Gifuni, A. J. , F. Pereira , M. M. Chakravarty , et al. 2024. “Perception of Social Inclusion/Exclusion and Response Inhibition in Adolescents With Past Suicide Attempt: A Multidomain Task‐Based fMRI Study.” Molecular Psychiatry 29, no. 7: 2135–2144. 10.1038/s41380-024-02485-w. 38424142

[psyp70081-bib-0046] Goldstein, D. S. 2021. “Stress and the ‘Extended’ Autonomic System.” Autonomic Neuroscience 236: 102889. 10.1016/j.autneu.2021.102889. 34656967 PMC10699409

[psyp70081-bib-0047] Gray, J. A. , and N. McNaughton . 2000. The Neuropsychology of Anxiety: An Enquiry in to the Functions of the Septo‐Hippocampal System. 2nd ed. Oxford University Press.

[psyp70081-bib-0048] Gunther Moor, B. , E. A. Crone , and M. W. van der Molen . 2010. “The Heartbrake of Social Rejection: Heart Rate Deceleration in Response to Unexpected Peer Rejection.” Psychological Science 21, no. 9: 1326–1333. 10.1177/095679761037923. 20696852

[psyp70081-bib-0049] IJzerman, H. , M. Gallucci , W. T. Pouw , S. C. Weiβgerber , N. J. Van Doesum , and K. D. Williams . 2012. “Cold‐Blooded Loneliness: Social Exclusion Leads to Lower Skin Temperatures.” Acta Psychologica 140, no. 3: 283–288. 10.1016/j.actpsy.2012.05.002. 22717422

[psyp70081-bib-0050] Ioannou, S. , H. Chotard , and M. Davila‐Ross . 2015. “No Strings Attached: Physiological Monitoring of Rhesus Monkeys ( *Macaca mulatta* ) With Thermal Imaging.” Frontiers in Behavioral Neuroscience 9: 160. 10.3389/fnbeh.2015.00160. 26150774 PMC4472989

[psyp70081-bib-0051] Ioannou, S. , S. Ebisch , T. Aureli , et al. 2013. “The Autonomic Signature of Guilt in Children: A Thermal Infrared Imaging Study.” PLoS One 8, no. 11: e79440. 10.1371/journal.pone.0079440. 24260220 PMC3834185

[psyp70081-bib-0052] Ioannou, S. , V. Gallese , and A. Merla . 2014. “Thermal Infrared Imaging in Psychophysiology: Potentialities and Limits.” Psychophysiology 51, no. 10: 951–963. 10.1111/psyp.12243. 24961292 PMC4286005

[psyp70081-bib-0053] Ioannou, S. , P. H. Morris , M. Baker , V. Reddy , and V. Gallese . 2017. “Seeing a Blush on the Visible and Invisible Spectrum: A Functional Thermal Infrared Imaging Study.” Frontiers in Human Neuroscience 11: 525. 10.3389/fnhum.2017.00525. 29163105 PMC5675873

[psyp70081-bib-0054] Jamal, S. K. M. , and E. Kamioka . 2019. “Emotions Detection Scheme Using Facial Skin Temperature and Heart Rate Variability.” In MATEC Web of Conferences, vol. 277, 02037. EDP Sciences. 10.1051/matecconf/201927702037.

[psyp70081-bib-0055] Jentsch, V. L. , and O. T. Wolf . 2020. “The Impact of Emotion Regulation on Cardiovascular, Neuroendocrine and Psychological Stress Responses.” Biological Psychology 154: 107893. 10.1016/j.biopsycho.2020.107893. 32437903

[psyp70081-bib-0056] Kang, J. , J. A. McGinley , G. McFadyen , and K. Babski‐Reeves . 2006. “Determining Learning Level and Effective Training Times Using Thermography.” In Proceedings of Army Science Conference, Orlando, Florida, USA.

[psyp70081-bib-0057] Kassam, K. S. , and W. B. Mendes . 2013. “The Effects of Measuring Emotion: Physiological Reactions to Emotional Situations Depend on Whether Someone Is Asking.” PLoS One 8, no. 6: e64959. 10.1371/journal.pone.0064959. 23785407 PMC3680163

[psyp70081-bib-0058] Kassner, M. P. , E. D. Wesselmann , A. T. Law , and K. D. Williams . 2012. “Virtually Ostracized: Studying Ostracism in Immersive Virtual Environments.” Cyberpsychology, Behavior, and Social Networking 15, no. 8: 399–403. 10.1089/cyber.2012.0113. 22897472 PMC3422048

[psyp70081-bib-0059] Kaushik, R. M. , R. Kaushik , S. K. Mahajan , and V. Rajesh . 2006. “Effects of Mental Relaxation and Slow Breathing in Essential Hypertension.” Complementary Therapies in Medicine 14, no. 2: 120–126. 10.1016/j.ctim.2005.11.007. 16765850

[psyp70081-bib-0060] Kellogg, D. L., Jr. 2006. “In Vivo Mechanisms of Cutaneous Vasodilation and Vasoconstriction in Humans During Thermoregulatory Challenges.” Journal of Applied Physiology 100, no. 5: 1709–1718. 10.1152/japplphysiol.01071.2005. 16614368

[psyp70081-bib-0061] Kelly, M. , S. McDonald , and J. Rushby . 2012. “All Alone With Sweaty Palms—Physiological Arousal and Ostracism.” International Journal of Psychophysiology 83, no. 3: 309–314. 10.1016/j.ijpsycho.2011.11.008. 22130492

[psyp70081-bib-0062] Khan, M. M. , M. Ingleby , and R. D. Ward . 2006. “Automated Facial Expression Classification and Affect Interpretation Using Infrared Measurement of Facial Skin Temperature Variations.” ACM Transactions on Autonomous and Adaptive Systems (TAAS) 1, no. 1: 91–113. 10.1145/1152934.1152939.

[psyp70081-bib-0063] Khan, M. M. , R. D. Ward , and M. Ingleby . 2009. “Classifying Pretended and Evoked Facial Expressions of Positive and Negative Affective States Using Infrared Measurement of Skin Temperature.” ACM Transactions on Applied Perception 6, no. 1: 1–22.

[psyp70081-bib-0064] Kim, H. G. , E. J. Cheon , D. S. Bai , Y. H. Lee , and B. H. Koo . 2018. “Stress and Heart Rate Variability: A Meta‐Analysis and Review of the Literature.” Psychiatry Investigation 15, no. 3: 235–245. 10.30773/pi.2017.08.17. 29486547 PMC5900369

[psyp70081-bib-0065] Kip, A. , T. M. Erle , W. W. Sleegers , and I. van Beest . 2025. “Choice Availability and Incentive Structure Determine How People Cope With Ostracism.” Journal of Experimental Social Psychology 117: 104707. 10.1016/j.jesp.2024.104707.

[psyp70081-bib-0066] Kopaczka, M. , K. Acar , and D. Merhof . 2016. “Robust Facial Landmark Detection and Face Tracking in Thermal Infrared Images Using Active Appearance Models.” In Joint Conference on Computer Vision, Imaging and Computer Graphics Theory and Applications, 150–158. VISIGRAPP. 10.5220/0005716801500158.

[psyp70081-bib-0067] Kopaczka, M. , R. Kolk , and D. Merhof . 2018. “A Fully Annotated Thermal Face Database and Its Application for Thermal Facial Expression Recognition.” In International Instrumentation and Measurement Technology Conference, 1–6. IEEE. 10.1109/I2MTC.2018.8409768.

[psyp70081-bib-0068] Kopaczka, M. , J. Nestler , and D. Merhof . 2017. “Face Detection in Thermal Infrared Images: A Comparison of Algorithm‐and Machine‐Learning‐Based Approaches.” In Advanced Concepts for Intelligent Vision Systems, 518–529. Springer. 10.1007/978-3-319-70353-4_44.

[psyp70081-bib-0069] Kosonogov, V. , L. De Zorzi , J. Honore , et al. 2017. “Facial Thermal Variations: A New Marker of Emotional Arousal.” PLoS One 12, no. 9: e0183592. 10.1371/journal.pone.0183592. 28922392 PMC5603162

[psyp70081-bib-0070] Kouchaki, M. , and J. Wareham . 2015. “Excluded and Behaving Unethically: Social Exclusion, Physiological Responses, and Unethical Behavior.” Journal of Applied Psychology 100, no. 2: 547–556. 10.1037/a0038034. 25314369

[psyp70081-bib-0071] Krill, A. , and S. M. Platek . 2009. “In‐Group and Out‐Group Membership Mediates Anterior Cingulate Activation to Social Exclusion.” Frontiers in Evolutionary Neuroscience 1: 438. 10.3389/neuro.18.001.2009. PMC2704010 19597546

[psyp70081-bib-0072] Kuraoka, K. , and K. Nakamura . 2011. “The Use of Nasal Skin Temperature Measurements in Studying Emotion in Macaque Monkeys.” Physiology & Behavior 102, no. 3–4: 347–355. 10.1016/j.physbeh.2010.11.029. 21130103

[psyp70081-bib-0073] Kuznetsova, A. , P. B. Brockhoff , and R. H. B. Christensen . 2017. “lmerTest Package: Tests in Linear Mixed Effects Models.” Journal of Statistical Software 82, no. 13: 1–26. 10.18637/jss.v082.i13.

[psyp70081-bib-0074] Lazarus, R. S. 1991. Emotion and Adaptation. Oxford University Press.

[psyp70081-bib-0075] Lazarus, R. S. , and S. Folkman . 1984. Stress, Appraisal, and Coping. Springing Publishing Company, Inc.

[psyp70081-bib-0076] Leliveld, M. C. , E. van Dijk , and I. van Beest . 2012. “Punishing and Compensating Others at Your Own Expense: The Role of Empathic Concern on Reactions to Distributive Injustice.” European Journal of Social Psychology 42, no. 2: 135–140. 10.1002/ejsp.872.

[psyp70081-bib-0077] Levenson, R. W. 1994. “The Nature of Emotion: Fundamental Questions.” In Human Emotions: A Functional View, edited by P. Ekman and R. J. Davidson , 123–126. Oxford University Press.

[psyp70081-bib-0079] Magnon, V. , F. Dutheil , and G. T. Vallet . 2021. “Benefits From One Session of Deep and Slow Breathing on Vagal Tone and Anxiety in Young and Older Adults.” Scientific Reports 11, no. 1: 19267. 10.1038/s41598-021-98736-9. 34588511 PMC8481564

[psyp70081-bib-0080] Maner, J. K. , C. N. DeWall , R. F. Baumeister , and M. Schaller . 2007. “Does Social Exclusion Motivate Interpersonal Reconnection? Resolving the ‘Porcupine Problem’.” Journal of Personality and Social Psychology 92, no. 1: 42–55. 10.1037/0022-3514.92.1.42. 17201541

[psyp70081-bib-0081] Masten, C. L. , S. A. Morelli , and N. I. Eisenberger . 2011. “An fMRI Investigation of Empathy for ‘Social Pain’ and Subsequent Prosocial Behavior.” NeuroImage 55, no. 1: 381–388. 10.1016/j.neuroimage.2010.11.060. 21122817

[psyp70081-bib-0082] Mauss, I. B. , R. W. Levenson , L. McCarter , F. H. Wilhelm , and J. J. Gross . 2005. “The Tie That Binds? Coherence Among Emotion Experience, Behavior, and Physiology.” Emotion 5, no. 2: 175–190. 10.1037/1528-3542.5.2.175. 15982083

[psyp70081-bib-0083] Mazzone, A. , M. Camodeca , D. Cardone , and A. Merla . 2017. “Bullying Perpetration and Victimization in Early Adolescence: Physiological Response to Social Exclusion.” International Journal of Developmental Science 11, no. 3–4: 121–130. 10.3233/DEV-170225.

[psyp70081-bib-0084] McFarland, R. A. , and R. Kadish . 1991. “Sex Differences in Finger Temperature Response to Music.” International Journal of Psychophysiology 11, no. 3: 295–298. 10.1016/0167-8760(91)90024-R. 1797764

[psyp70081-bib-0086] Meral, E. O. , H. Rosenbusch , A. Kip , D. Ren , E. van Dijk , and I. van Beest . 2022. “Social Ball: An Immersive Research Paradigm to Study Social Ostracism [Preprint].” 10.31234/osf.io/v8kwx.

[psyp70081-bib-0087] Naemura, A. , K. Tsuda , and N. Suzuki . 1993. “Effects of Loud Noise on Nasal Skin Temperature.” Shinrigaku Kenkyu: The Japanese Journal of Psychology 64, no. 1: 51–54. 10.4992/jjpsy.64.51. 8355430

[psyp70081-bib-0088] Nakayama, K. , S. Goto , K. Kuraoka , and K. Nakamura . 2005. “Decrease in Nasal Temperature of Rhesus Monkeys ( *Macaca mulatta* ) in Negative Emotional State.” Physiology & Behavior 84, no. 5: 783–790. 10.1016/j.physbeh.2005.03.009. 15885256

[psyp70081-bib-0089] Nhan, B. R. , and T. Chau . 2009. “Classifying Affective States Using Thermal Infrared Imaging of the Human Face.” IEEE Transactions on Biomedical Engineering 57, no. 4: 979–987. 10.1109/tbme.2009.2035926. 19923040

[psyp70081-bib-0090] Nozawa, A. , and M. Tacano . 2009. “Correlation Analysis on Alpha Attenuation and Nasal Skin Temperature.” Journal of Statistical Mechanics: Theory and Experiment 2009: P01007. 10.1088/1742-5468/2009/01/P01007.

[psyp70081-bib-0091] Oken, B. S. , I. Chamine , and W. Wakeland . 2015. “A Systems Approach to Stress, Stressors and Resilience in Humans.” Behavioural Brain Research 282: 144–154. 10.1016/j.bbr.2014.12.047. 25549855 PMC4323923

[psyp70081-bib-0092] Or, C. K. L. , and V. G. Duffy . 2007. “Development of a Facial Skin Temperature‐Based Methodology for Non‐Intrusive Mental Workload Measurement.” Occupational Ergonomics 7, no. 2: 83–94. 10.3233/oer-2007-7202.

[psyp70081-bib-0093] Panasiti, M. S. , D. Cardone , E. F. Pavone , A. Mancini , A. Merla , and S. M. Aglioti . 2016. “Thermal Signatures of Voluntary Deception in Ecological Conditions.” Scientific Reports 6, no. 1: 35174. 10.1038/srep35174. 27734927 PMC5062078

[psyp70081-bib-0094] Panasiti, M. S. , P. Giorgia , B. Monachesi , L. Lorenzini , V. Panasiti , and S. M. Aglioti . 2019. “Cognitive Load and Emotional Processing in Psoriasis: A Thermal Imaging Study.” Experimental Brain Research 237, no. 1: 211–222. 10.1007/s00221-018-5416-y. 30374785

[psyp70081-bib-0095] Paolini, D. , F. R. Alparone , D. Cardone , I. van Beest , and A. Merla . 2016. “‘The Face of Ostracism’: The Impact of the Social Categorization on the Thermal Facial Responses of the Target and the Observer.” Acta Psychologica 163: 65–73. 10.1016/j.actpsy.2015.11.001. 26613387

[psyp70081-bib-0096] Parsons, T. D. 2015. “Virtual Reality for Enhanced Ecological Validity and Experimental Control in the Clinical, Affective and Social Neurosciences.” Frontiers in Human Neuroscience 9: 660. 10.3389/fnhum.2015.00660. 26696869 PMC4675850

[psyp70081-bib-0097] Ponsi, G. , B. Monachesi , V. Panasiti , S. M. Aglioti , and M. S. Panasiti . 2019. “Physiological and Behavioral Reactivity to Social Exclusion: A Functional Infrared Thermal Imaging Study in Patients With Psoriasis.” Journal of Neurophysiology 121, no. 1: 38–49. 10.1152/jn.00555.2018. 30379630 PMC6383668

[psyp70081-bib-0099] Ren, D. , E. Wesselmann , and K. D. Williams . 2016. “Evidence for Another Response to Ostracism: Solitude Seeking.” Social Psychological and Personality Science 7, no. 3: 204–212. 10.1177/1948550615616169.

[psyp70081-bib-0100] Ren, D. , E. D. Wesselmann , and I. van Beest . 2021. “Seeking Solitude After Being Ostracized: A Replication and Beyond.” Personality and Social Psychology Bulletin 47, no. 3: 426–440. 10.1177/0146167220928238. 32515281 PMC7897794

[psyp70081-bib-0101] Ren, D. , E. D. Wesselmann , and K. D. Williams . 2018. “Hurt People Hurt People: Ostracism and Aggression.” Current Opinion in Psychology 19: 34–38. 10.1016/j.copsyc.2017.03.026. 29279219

[psyp70081-bib-0102] Rimm‐Kaufman, S. E. , and J. Kagan . 1996. “The Psychological Significance of Changes in Skin Temperature.” Motivation and Emotion 20, no. 1: 63–78. 10.1007/BF02251007.

[psyp70081-bib-0103] Rotge, J. Y. , C. Lemogne , S. Hinfray , et al. 2015. “A Meta‐Analysis of the Anterior Cingulate Contribution to Social Pain.” Social Cognitive and Affective Neuroscience 10, no. 1: 19–27. 10.1093/scan/nsu110. 25140048 PMC4994851

[psyp70081-bib-0104] Roth, S. , and L. J. Cohen . 1986. “Approach, Avoidance, and Coping With Stress.” American Psychologist 41, no. 7: 813–819. 10.1037/0003-066X.41.7.813. 3740641

[psyp70081-bib-0105] Salazar‐López, E. , E. Domínguez , V. J. Ramos , et al. 2015. “The Mental and Subjective Skin: Emotion, Empathy, Feelings and Thermography.” Consciousness and Cognition 34: 149–162. 10.1016/j.concog.2015.04.003. 25955182

[psyp70081-bib-0107] Sijtsema, J. J. , E. K. Shoulberg , and D. Murray‐Close . 2011. “Physiological Reactivity and Different Forms of Aggression in Girls: Moderating Roles of Rejection Sensitivity and Peer Rejection.” Biological Psychology 86, no. 3: 181–192. 10.1016/j.biopsycho.2010.11.007. 21129435

[psyp70081-bib-0108] Sleegers, W. W. , T. Proulx , and I. van Beest . 2017. “The Social Pain of Cyberball: Decreased Pupillary Reactivity to Exclusion Cues.” Journal of Experimental Social Psychology 69: 187–200. 10.1016/j.jesp.2016.08.004.

[psyp70081-bib-0109] Smart Richman, L. , and M. R. Leary . 2009. “Reactions to Discrimination, Stigmatization, Ostracism, and Other Forms of Interpersonal Rejection: A Multi‐Motive Model.” Psychological Review 116, no. 2: 365–383. 10.1037/a0015250. 19348546 PMC2763620

[psyp70081-bib-0110] Sonkusare, S. , D. Ahmedt‐Aristizabal , M. J. Aburn , et al. 2019. “Detecting Changes in Facial Temperature Induced by a Sudden Auditory Stimulus Based on Deep Learning‐Assisted Face Tracking.” Scientific Reports 9, no. 1: 4729. 10.1038/s41598-019-41172-7. 30894584 PMC6426955

[psyp70081-bib-0111] Sunami, N. , M. A. Nadzan , and L. M. Jaremka . 2019. “The Bi‐Dimensional Rejection Taxonomy: Organizing Responses to Social Rejection Along Antisocial–Prosocial and Engaged–Disengaged Dimensions.” Social and Personality Psychology Compass 13, no. 9: e12497. 10.1111/spc3.12497.

[psyp70081-bib-0113] Van Beest, I. , and W. Sleegers . 2019. “Physiostracism: A Case for Non‐Invasive Measures of Arousal in Ostracism Research.” In Current Directions in Ostracism, Social Exclusion and Rejection Research, edited by S. C. Rudert , R. Greifeneder , and K. D. Williams , 120–135. Routledge.

[psyp70081-bib-0114] Van Beest, I. , and K. D. Williams . 2006. “When Inclusion Costs and Ostracism Pays, Ostracism Still Hurts.” Journal of Personality and Social Psychology 91, no. 5: 918–928. 10.1037/0022-3514.91.5.918. 17059310

[psyp70081-bib-0115] Veltman, H. , and W. Vos . 2005. “Facial Temperature as a Measure of Operator State.” In Foundations of Augmented Cognition, edited by D. D. Schmorrow , 293–301. CRC Press Taylor & Francis Group.

[psyp70081-bib-0116] Watson, D. , L. A. Clark , and A. Tellegen . 1988. “Development and Validation of Brief Measures of Positive and Negative Affect: The PANAS Scales.” Journal of Personality and Social Psychology 54, no. 6: 1063–1070. 10.1037/0022-3514.54.6.1063. 3397865

[psyp70081-bib-0117] Wehrwein, E. , H. Orer , and S. Barman . 2016. “Overview of the Anatomy, Physiology, and Pharmacology of the Autonomic Nervous.” Comprehensive Physiology 6, no. 3: 1239–1278. 10.1002/cphy.c150037. 27347892

[psyp70081-bib-0118] Wesley, N. O. , and H. I. Maibach . 2003. “Racial (Ethnic) Differences in Skin Properties: The Objective Data.” American Journal of Clinical Dermatology 4, no. 12: 843–860. 10.2165/00128071-200304120-00004. 14640777

[psyp70081-bib-0119] Wesselmann, E. D. , D. Ren , and K. D. Williams . 2015. “Motivations for Responses to Ostracism.” Frontiers in Psychology 6: 40. 10.3389/fpsyg.2015.00040. 25691876 PMC4315012

[psyp70081-bib-0120] Williams, K. D. 2007. “Ostracism.” Annual Review of Psychology 58, no. 1: 425–452. 10.1146/annurev.psych.58.110405.085641. 16968209

[psyp70081-bib-0121] Williams, K. D. 2009. “Ostracism: A Temporal Need‐Threat Model.” In Advances in Experimental Social Psychology, edited by M. P. Zanna , vol. 41, 275–314. ScienceDirect. 10.1016/S0065-2601(08)00406-1.

[psyp70081-bib-0122] Williams, K. D. , and B. Jarvis . 2006. “Cyberball: A Program for Use in Research on Interpersonal Ostracism and Acceptance.” Behavior Research Methods 38, no. 1: 174–180. 10.3758/BF03192765. 16817529

[psyp70081-bib-0123] Williams, K. D. , and S. A. Nida . 2011. “Ostracism: Consequences and Coping.” Current Directions in Psychological Science 20, no. 2: 71–75. 10.1177/0963721411402480.

[psyp70081-bib-0124] Wirth, J. , F. Feldberg , A. P. Schouten , B. van den Hooff , and K. D. Williams . 2011. “Using Virtual Game Environments to Study Group Behaviour.” In Research Methods for Studying Groups and Teams: A Guide to Approaches, Tools, and Technologies, edited by A. Hollingshead and M. S. Poole , 1–24. Routledge.

[psyp70081-bib-0125] Yuan, J. , N. Ding , Y. Liu , and J. Yang . 2015. “Unconscious Emotion Regulation: Nonconscious Reappraisal Decreases Emotion‐Related Physiological Reactivity During Frustration.” Cognition and Emotion 29, no. 6: 1042–1053. 10.1080/02699931.2014.965663. 25297822

