# Supplementary material for: Facial Temperature Responses to Ostracism in Women: Exploring Nasal Thermal Signatures of Different Coping Behaviors
Source: Psychophysiology. 2025 Jun 8;62(6):e70081. doi: 10.1111/psyp.70081 (PMC12146686; doi:10.1111/psyp.70081)
Supplement: Supplementary file 1 — Data S1. [file PSYP-62-e70081-s001.pdf]

Supporting information 1

Participants subsample

As pre-registered, we also report the main analyses without participants who indicated that they received <= 20% of the ball-tosses during the inclusion condition (*n* = 2), and/or indicated that they received >= 26% of the ball-tosses during the ostracism condition (*n* = 0), and/or did not recall the consequences of their behavioral response correctly (*n* = 0). After exclusion, a subsample of 93 participants remained included with an average age of 20.27 (*SD* = 1.90) for the analyses reported below.

Manipulation checks self-reported measures

Four separate paired t-tests (one-sided) were conducted as manipulation checks to assess participants' experiences during the two Cyberball conditions based on the following self-reported measures: The perceived percentage of received ball-tosses was lower following ostracism (*M* = 1.31, *SD* = 2.22) compared to inclusion (*M* = 36.08, *SD* = 10.16), $t(92) = 32.02$, $p < .001$, $d = 3.32$. Need satisfaction was lower following ostracism (*M* = 1.87, *SD* = 0.64) compared to inclusion (*M* = 5.37, *SD* = 0.94), $t(92) = 28.63$, $p < .001$, $d = 2.97$. Negative affect was higher following ostracism (*M* = 4.31, *SD* = 1.32) compared to inclusion (*M* = 1.55, *SD* = 0.69), $t(92) = -19.51$, $p < .001$, $d = -2.02$. Positive affect was lower following ostracism (*M* = 1.68, *SD* = 10.78) compared to inclusion (*M* = 3.90, *SD* = 1.61), $t(92) = 13.08$, $p < .001$, $d = 1.36$. Consistent with the results reported in the main manuscript, the manipulation checks based on these self-reported measures were deemed successful.

Main analyses: Behavioral responses to ostracism

A total of 49 participants chose to withdraw (do nothing) during the hypothetical Allocation game and 17 participants allocated coins to respond prosocial (*M* = 4.47, *SD* = *3.45*). After applying the exclusion criteria, only 27 instead of 29 participants allocated coins to respond antisocial (*M* = 3.75, *SD* = 2.03).

More participants chose to respond antisocial (29.03%) compared to prosocial (18.28%) after experiencing ostracism. However, this difference was not significant, odds = 1.59, 95% CI [0.87, 2.91], $p = .135$. Participants were more likely to withdraw (52.69%) after ostracism than to act prosocially, odds = 2.88, 95% CI [1.66, 5.00], $p < .001$, and showed a greater tendency to choose withdrawal over antisocial responses, odds = 1.82, 95% CI [1.14, 2.90], $p = .013$. Overall, the results on the behavioral responses to ostracism remained consistent with the results reported in the main manuscript.

Main analyses: Facial Cutaneous Temperature Changes in Response to Ostracism

The results for the main analyses on facial cutaneous temperature changes in response to ostracism as well as the results for the exploratory analyses on temperature changes of the nose region in relation to behavioral responses to ostracism remained consistent with those in the main manuscript. For reasons of conciseness and to avoid redundancy, we do not report the full analyses again here. Researchers interested in conducting additional analyses on the subsample, from which two participants were excluded based on predetermined criteria, can do so using the code provided on OSF: https://osf.io/mhc67/?view_only=cfbd4fe4eec64d888ba03e57c594d8df. Specifically, the subsample used for these analyses excludes the two participants removed according to the exclusion criteria.
