# Supplementary material for: Facial Temperature Responses to Ostracism in Women: Exploring Nasal Thermal Signatures of Different Coping Behaviors
Source: Psychophysiology. 2025 Jun 8;62(6):e70081. doi: 10.1111/psyp.70081 (PMC12146686; doi:10.1111/psyp.70081)
Supplement: Supplementary file 2 — Data S2. [file PSYP-62-e70081-s002.pdf]

Supporting information 2

Facial Cutaneous Temperature During Baseline Assessments

Deviating from our pre-registration, we performed additional analyses to explore potential differences in absolute facial temperature values during the two baseline periods recorded prior to the inclusion and ostracism conditions of the hypothetical Cyberball games. The goal of these additional analyses was to ensure that any effects observed in facial temperature changes during the experimental conditions could not be explained by potential temporal or environmental influences.

Specifically, we performed a linear mixed-effects model, to test whether participants showed different facial cutaneous temperature values during the baseline assessment before inclusion compared to the baseline before ostracism, whether potential differences in facial cutaneous temperature changes between baseline conditions were different over time. In this analysis, the variable 'time' refers to the 10 equally time-spaced frames selected throughout each baseline recording. The model included fixed effects of time, baseline condition, and facial region of interest (ROI) and all their interactions, the random intercepts for each subject, as well as random slopes for condition within each subject. The results are reported in Table S4-1 and shown in Figure S4-1.

Overall, these findings indicate that facial temperature during the two baseline periods was stable and not significantly affected by environmental or temporal variables, and that regional temperature patterns remained consistent across the baseline conditions.

Table S2-1

Baseline Model Output: Type III analysis of Variance Table for the Fixed Effects with Satterthwaite's Method

Fixed effects	***SS***	***MS***	***F***	***df***	***p***	p_{FDR}
Time	3.4	3.43	1.86	(1,12328.0)	.172	.603
Baseline	1.0	1.00	0.55	(1, 547.9)	.461	.883
ROI	7510.7	1072.95	583.68	(7, 12304.7)	**< .001**	**< .001**
Time*Baseline	0.4	0.43	0.23	(1, 12346.7)	.631	.883
Time*ROI	11.1	1.59	0.87	(7, 12303.5)	.533	.883
Baseline*ROI	5.4	0.77	0.42	(7, 12310.2)	.892	.974
Time*baseline*ROI	3.1	0.45	0.25	(7, 12304.2)	.974	.974

Note. Ref. category condition: baseline inclusion, number of observations: 12501, groups: subject, 94.

p_{FDR} = false discovery rate correction applied to the seven *p*-values for the fixed effects reported in Table S2-1.

Figure S2-1

The Fixed Effects of Time and Baseline Condition on Absolute Temperature Values °C for each Facial Region of Interest (ROI)

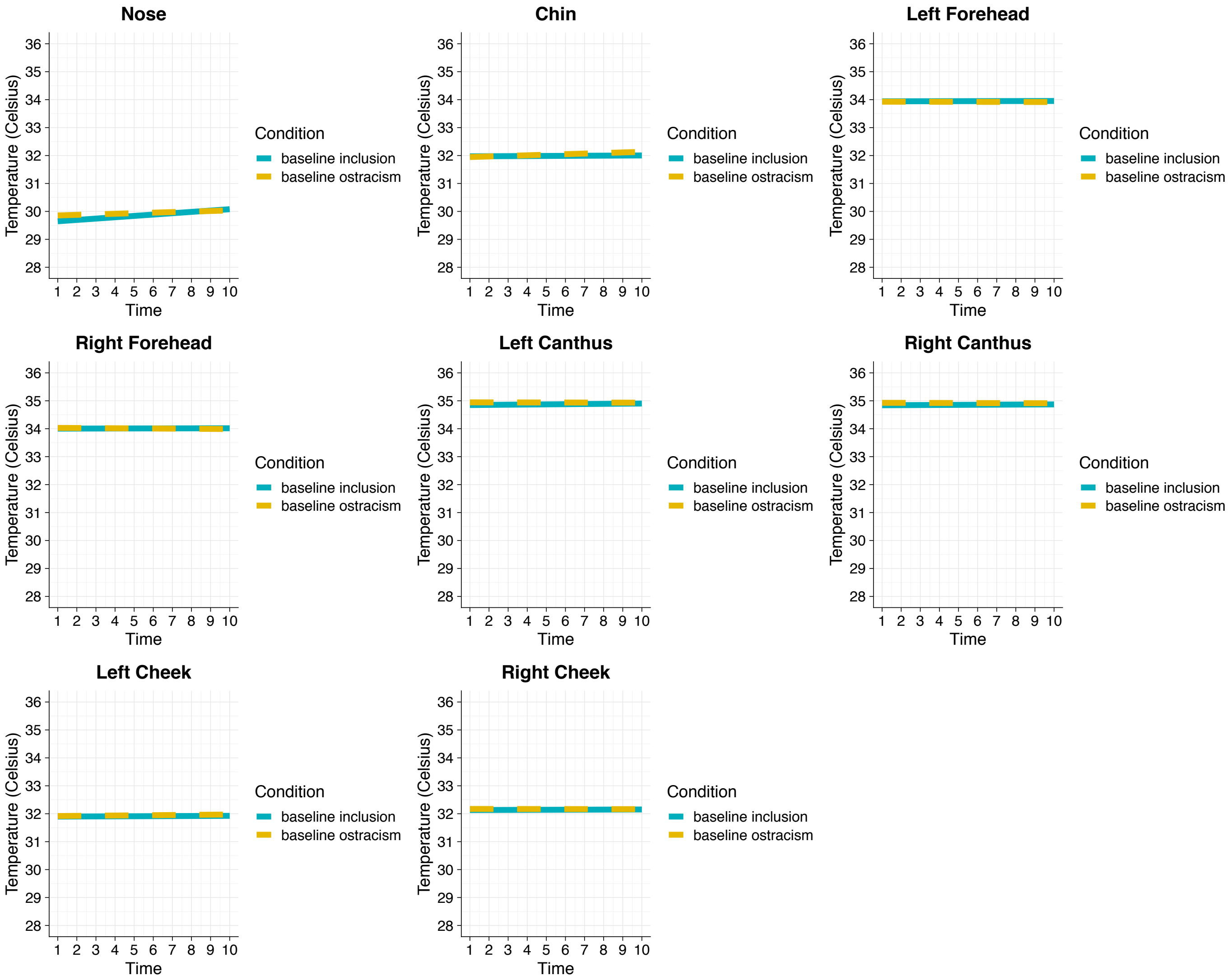

Note. Plots derived from summarized data (i.e., not model estimates).
