# Supplementary material for: Facial Temperature Responses to Ostracism in Women: Exploring Nasal Thermal Signatures of Different Coping Behaviors
Source: Psychophysiology. 2025 Jun 8;62(6):e70081. doi: 10.1111/psyp.70081 (PMC12146686; doi:10.1111/psyp.70081)
Supplement: Supplementary file 3 — Data S3. [file PSYP-62-e70081-s004.pdf]

Supporting information 3

Table S3-1

Maximal Model Output (before stepwise elimination): Type III analysis of Variance Table for the Fixed Effects with Satterthwaite's Method

**Fixed effects**	**SS**	**MS**	***F***	***df***	***p***	p_{FDR}
Time	4.15	4.15	27.24	(1, 137.6)	< **.001**	< **.001**
Condition	0.06	0.06	0.39	(1, 96.7)	.532	.667
ROI	30.11	4.30	28.23	(7, 11321.5)	< **.001**	< **.001**
Time*Condition	0.05	0.05	0.32	(1, 105.5)	.572	.667
Time*ROI	20.57	2.94	19.28	(7, 11319.3)	< **.001**	< **.001**
Condition*ROI	2.37	0.34	2.23	(7, 11313.1)	.029	.051
Time*Condition*ROI	0.51	0.07	0.47	(7, 11306.6)	.854	.854

Note. p_{FDR} = false discovery rate correction applied to the seven *p*-values for the fixed effects reported in Table S3-1.

Table S3-2

Model Output (after stepwise elimination): Type III analysis of Variance Table for the Fixed Effects with Satterthwaite's Method

**Fixed effects**	**SS**	**MS**	***F***	***df***	***p***	***p_{FDR}***
Time	4.29	4.29	28.11	(1, 176.5)	< **.001**	< **.001**
Condition	0.18	0.18	1.16	(1, 91.9)	.285	.285
ROI	30.27	4.34	28.35	(7, 11394.1)	< **.001**	< **.001**
Time*ROI	20.49	2.93	19.19	(7, 11394.2)	< **.001**	< **.001**
Condition*ROI	7.20	1.03	6.75	(7, 11404.8)	**<.001**	**<.001**

Note. p_{FDR} = false discovery rate correction applied to the seven *p*-values for the fixed effects reported in Table S3-2.
