# Supplementary material for: Facial Temperature Responses to Ostracism in Women: Exploring Nasal Thermal Signatures of Different Coping Behaviors
Source: Psychophysiology. 2025 Jun 8;62(6):e70081. doi: 10.1111/psyp.70081 (PMC12146686; doi:10.1111/psyp.70081)
Supplement: Supplementary file 4 — Data S4. [file PSYP-62-e70081-s003.pdf]

Supporting information 4

Table S4-1

Final Model Output: All Interaction Effects Between Time and Facial Regions of Interest (ROIs), Contrasting Each Region Against Another

**Fixed effects Time:ROI**	***b***	***SE***	***df***	***t*-value**	***p*-value**	***p*-value (FDR)**
Time:ROI (nose: chin)	0.034	0.006	11399.40	6.19	< **.001**	< **.001**
Time:ROI (nose: lhead)	0.037	0.005	11395.37	7.27	< **.001**	< **.001**
Time:ROI (nose: rhead)	0.037	0.005	11395.13	7.27	< **.001**	< **.001**
Time:ROI (nose: lcanthus)	0.042	0.005	11392.67	8.31	< **.001**	< **.001**
Time:ROI (nose: rcanthus)	0.043	0.005	11392.95	8.56	< **.001**	< **.001**
Time:ROI (nose: lcheek)	0.052	0.005	11391.66	10.26	< **.001**	< **.001**
Time:ROI (nose: rcheek)	0.047	0.005	11392.01	9.29	< **.001**	< **.001**
Time:ROI (chin: lhead)	0.003	0.005	11399.47	0.550	.582	.632
Time:ROI (chin: rhead)	0.003	0.005	11399.40	0.544	.586	.632
Time:ROI (chin:lcantus)	0.008	0.005	11400.48	1.451	.147	.242
Time:ROI (chin:rcanthus)	0.009	0.005	11401.21	1.682	.093	.162
Time:ROI (chin:lcheek)	0.018	0.005	11401.58	3.361	< **.001**	**.003**
Time:ROI (chin:rcheek)	0.013	0.005	11401.76	2.400	.016	.042
Time:ROI (lhead:rhead)	-0.000	0.005	11389.28	-0.008	.994	.994
Time:ROI (lhead:lcantus)	0.005	0.005	11393.64	0.970	.332	.404
Time:ROI (lhead:rcanthus)	0.006	0.005	11393.55	1.220	.223	.328
Time:ROI (lhead:lcheek)	0.015	0.005	11394.42	3.043	**.002**	**.007**
Time:ROI (lhead:rcheek)	0.010	0.005	11393.81	2.001	.045	.091
Time:ROI (rhead:lcanthus)	0.005	0.005	11393.47	0.981	.327	.404
Time:ROI (rhead:rcanthus)	0.006	0.005	11393.31	1.231	.218	.328
Time:ROI (rhead:lcheek)	0.015	0.005	11394.18	3.058	**.002**	**.007**
Time:ROI (rhead:rcheek)	0.010	0.005	11393.47	2.014	.044	.091
Time:ROI (lcanthus:rcanthus)	0.001	0.005	11389.40	0.252	.801	.831
Time:ROI (lcanthus:lcheek)	0.011	0.005	11392.10	2.111	.035	.081
Time:ROI (lcanthus:rcheek)	0.005	0.004	11391.94	1.049	.294	.392
Time:ROI (rcanthus:lcheek)	0.009	0.005	11391.79	1.866	.062	.116
Time:ROI (rcanthus:rcheek)	0.004	0.005	11391.69	0.800	.424	.494
Time:ROI (lcheek:rcheek)	-0.005	0.005	11390.53	-1.066	.287	.392

Note. Number of observations: 11587, groups: subject, 94.

p_{FDR} = false discovery rate correction was applied to the twenty-eight *p*-values reported in Table S4-1.

Table S4-2

Final Model Output: All Interaction Effects Between Condition and Facial Regions of Interest (ROIs), Contrasting Each Region Against Another

**Fixed effect of Condition (ROI)**	***b***	***SE***	***df***	***t*-value**	***p*-value**	***p*-value (FDR)**
Condition:ROI (nose:chin)	-0.111	0.032	11437.54	-3.49	< **.001**	< **.001**
Condition:ROI (nose:lhead)	-0.135	0.029	11399.12	-4.62	< **.001**	< **.001**
Condition:ROI (nose:rhead)	-0.164	0.029	11399.55	-5.61	< **.001**	< **.001**
Condition:ROI (nose:lcanthus)	-0.097	0.029	11402.46	-3.35	< **.001**	**.002**
Condition:ROI (nose:rcanthus)	-0.134	0.029	11403.20	-4.64	< **.001**	< **.001**
Condition:ROI (nose:lcheek)	-0.119	0.029	11401.60	-4.06	< **.001**	< **.001**
Condition:ROI (nose:rcheek)	-0.044	0.029	11403.33	-1.52	.129	.225
Condition:ROI (chin: lhead)	-0.024	0.031	11437.86	-0.759	.448	.560
Condition:ROI (chin: rhead)	-0.053	0.031	11437.46	-1.674	.094	.188
Condition:ROI (chin:lcantus)	0.014	0.031	11432.11	0.453	.651	.701
Condition:ROI (chin:rcanthus)	-0.023	0.031	11432.58	-0.739	.460	.560
Condition:ROI (chin:lcheek)	-0.008	0.032	11440.74	-0.249	.804	.833
Condition:ROI (chin:rcheek)	0.067	0.031	11442.16	2.132	.033	.077
Condition:ROI (lhead:rhead)	-0.029	0.029	11389.36	-1.001	.317	.444
Condition:ROI (lhead:lcantus)	0.038	0.029	11397.00	1.334	.182	.291
Condition:ROI (lhead:rcanthus)	0.001	0.028	11395.88	0.032	.975	.975
Condition:ROI (lhead:lcheek)	0.016	0.029	11396.88	0.556	.578	.667
Condition:ROI (lhead:rcheek)	0.091	0.029	11396.00	3.170	**.002**	**.005**
Condition:ROI (rhead:lcanthus)	0.067	0.028	11396.29	2.347	.019	.048
Condition:ROI (rhead:rcanthus)	0.030	0.028	11395.18	1.043	.297	.437
Condition:ROI (rhead:lcheek)	0.045	0.029	11396.85	1.555	.120	.224
Condition:ROI (rhead:rcheek)	0.119	0.029	11395.08	4.181	< **.001**	< **.001**
Condition:ROI (lcanthus:rcanthus)	-0.037	0.028	11390.00	-1.320	.187	.291
Condition:ROI (lcanthus:lcheek)	-0.022	0.029	11401.48	-0.769	.442	.560
Condition:ROI (lcanthus:rcheek)	0.053	0.028	11400.32	1.860	.063	.136
Condition:ROI (rcanthus:lcheek)	0.015	0.029	11399.88	0.531	.595	.667
Condition:ROI (rcanthus:rcheek)	0.090	0.028	11397.72	3.175	**.002**	**.005**
Condition:ROI (lcheek:rcheek)	0.075	0.029	11393.82	2.603	**.009**	.026

Note. Ref. category condition: inclusion, number of observations: 11587, groups: subject, 94.

p_{FDR} = false discovery rate correction was applied to the twenty-eight *p*-values reported in Table S4-2.
